# Drug-resilient Cancer Cell Phenotype Is Acquired via Polyploidization Associated with Early Stress Response Coupled to HIF2α Transcriptional Regulation

**DOI:** 10.1158/2767-9764.CRC-23-0396

**Published:** 2024-03-07

**Authors:** Christopher Carroll, Auraya Manaprasertsak, Arthur Boffelli Castro, Hilda van den Bos, Diana C.J. Spierings, René Wardenaar, Anuraag Bukkuri, Niklas Engström, Etienne Baratchart, Minjun Yang, Andrea Biloglav, Charlie K. Cornwallis, Bertil Johansson, Catharina Hagerling, Marie Arsenian-Henriksson, Kajsa Paulsson, Sarah R. Amend, Sofie Mohlin, Floris Foijer, Alan McIntyre, Kenneth J. Pienta, Emma U. Hammarlund

**Affiliations:** 1 Department of Experimental Medical Science, Lund University, Lund, Sweden.; 2 Lund Stem Cell Center (SCC), Lund University, Lund, Sweden.; 3 Lund University Cancer Center (LUCC), Lund University, Lund, Sweden.; 4 European Research Institute for the Biology of Ageing, University of Groningen, University Medical Centre Groningen, Groningen, the Netherlands.; 5 Division of Clinical Genetics, Department of Laboratory Medicine, Lund University, Lund, Sweden.; 6 Department of Biology, Lund University, Lund, Sweden.; 7 Department of Microbiology, Tumor and Cell Biology (MTC), Karolinska Institutet, Biomedicum, Stockholm, Sweden.; 8 Cancer Ecology Center, the Brady Urological Institute, Johns Hopkins University School of Medicine, Baltimore, Maryland.; 9 Division of Pediatrics, Department of Clinical Sciences, Lund University, Lund, Sweden.; 10 Hypoxia and Acidosis Group, Nottingham Breast Cancer Research Centre, School of Medicine, Biodiscovery Institute, University of Nottingham, Nottingham, United Kingdom.

## Abstract

Therapeutic resistance and recurrence remain core challenges in cancer therapy. How therapy resistance arises is currently not fully understood with tumors surviving via multiple alternative routes. Here, we demonstrate that a subset of cancer cells survives therapeutic stress by entering a transient state characterized by whole-genome doubling. At the onset of the polyploidization program, we identified an upregulation of key transcriptional regulators, including the early stress-response protein AP-1 and normoxic stabilization of HIF2α. We found altered chromatin accessibility, ablated expression of retinoblastoma protein (RB1), and enrichment of AP-1 motif accessibility. We demonstrate that AP-1 and HIF2α regulate a therapy resilient and survivor phenotype in cancer cells. Consistent with this, genetic or pharmacologic targeting of AP-1 and HIF2α reduced the number of surviving cells following chemotherapy treatment. The role of AP-1 and HIF2α in stress response by polyploidy suggests a novel avenue for tackling chemotherapy-induced resistance in cancer.

**Significance:**

In response to cisplatin treatment, some surviving cancer cells undergo whole-genome duplications without mitosis, which represents a mechanism of drug resistance. This study presents mechanistic data to implicate AP-1 and HIF2α signaling in the formation of this surviving cell phenotype. The results open a new avenue for targeting drug-resistant cells.

## Introduction

Metastatic cancer is a major threat to human health because of its frequent resistance to systemic cytotoxic therapy (1, 2). Resistance is generally attributed to genetic tumor cell heterogeneity and random chance by which at least one cancer cell can survive a particular therapy and give rise to a subsequent treatment-resistant clone (3–5). However, the mechanisms underlying the emergence of therapy resistance remain largely undefined. On one hand, the appearance of mutations can be fueled by genetic instability or aneuploidy (6–10). On the other hand, the increase of genomic content allows for added genetic diversity, plasticity, and adaptability (6, 9). A particularly dramatic change in genomic content occurs when cells undergo whole-genome doubling and become polyploid. Importantly, this polyploidy is seen transiently in organisms across the Tree of Life as a stress-response mechanism (11): Environmental stress has been observed to induce increased cellular size in plants, invertebrates, and vertebrates (12–14). Similarly, an increase in cell size has been found in a subset of cancer cells in response to stressors like chemotherapy, radiation, hypoxia, mitotic inhibitors, hyperthermia, or acidosis (15–21). However, how this transient state of polyploidy leads to cell survival remains unclear (22). We hypothesized that cancer cells might survive cytotoxic therapy via conserved pathways that converge on perturbing cell cycle control. Such a survival mechanism would represent yet another path to cancer cell resistance.

Previous investigations have shown that Burkitt lymphoma cells exposed to radiation underwent four endoreplications before depolyploidization and recovery of resistant offspring. Irradiated p53 mutant cells but not p53 wild-type cells exhibit these endocycles and RNA sequencing (RNA-seq) data showed stem cell markers were upregulated in polyploid cells (23). This reprogramming was partially preventable via Notch inhibition indicating multiple pathways are responsible (24). The prolonged time before emergence of proliferating progeny after polyploidy has led to hypotheses that the polyploid cells acquire a senescence phenotype that is required for polyploidy (25).

Here, we investigated the structural, genomic, transcriptional, and epigenetic mechanisms that facilitate survival in cancer cells treated with cytotoxic drugs. Using microscopy and single-cell whole-genome sequencing (scWGS), we found that a small fraction of cells survived cytotoxic therapy and that these demonstrated plasticity, having enlarged nuclei and cell size. This phenotype was accompanied by genome polyploidization and a pause in proliferation. By applying RNA-seq, we identified AP-1 members *JUN*, *FOS*, and *FOSL1* and *EPAS1* as important mediators of survival and examined their functional role using CRISPR/Cas9-mediated knockout (KO) or pharmacologic inhibition. Assay for Transposase-Accessible Chromatin Using Sequencing (ATAC-seq) of surviving cells demonstrated substantial changes in chromatin accessibility, particularly around the HIF2α locus, and around proteins regulating the cell cycle, including the retinoblastoma protein (RB1). In the progeny of surviving polyploid cells, these changes were reverted as they transitioned back into a proliferative state. We further showed that inhibition of AP-1 and HIF2α led to a reduction in cancer cell survival under drug treatment. These results suggest a novel avenue to manage chemotherapy-induced resistance in cancer.

## Materials and Methods

### Cell Culture

HCC-1806 (breast), MDA-MB-231 (breast), MCF7 (breast), and PC3 (prostate) cells were purchased from ATCC and CAL-51 (breast), LS174T (colon) from Creative Bioarray. U1690 (lung), 786-0 (kidney) were supplied by Dr. Sofie Mohlin, Lund University (Lund, Sweden). All cell lines were maintained in DMEM GlutaMAX (Thermo Fisher Scientific, #11594446), supplemented with 10% FBS (Thermo Fisher Scientific, #11550356) without penicillin/streptomycin and were *Mycoplasma* tested (MycoAlert, Lonza, #LT07-318) at regular intervals. Cells were maintained in a humidified incubator at 5% CO_2_ and 37°C. All cell lines were authenticated in 2023, using short tandem repeat profiling (Eurofins).

### Chemicals

Cells were treated with cisplatin (Sigma-Aldrich, #232120); the list of LD_50_ for each cell line is presented in Supplementary Table S1. For inhibition studies, the c-Fos/AP-1 inhibitor T-5224 (MedChemExpress, #HY-12270), the HIF2α inhibitor Belzutifan (PT2977; MedChemExpress, #HY-125840), and the Notch inhibitor PF-03084014 (MedChemExpress, #HY-15185) were used at the IC_50_ (10 nmol/L) for 72 hours in conjunction with cisplatin (26). Cisplatin was solubilized in PBS with 140 mmol/L NaCl at a stock concentration of 3 mmol/L. The inhibitors were solubilized in DMSO at a concentration of 10 mmol/L.

### Treatment

**FIGURE 1:**
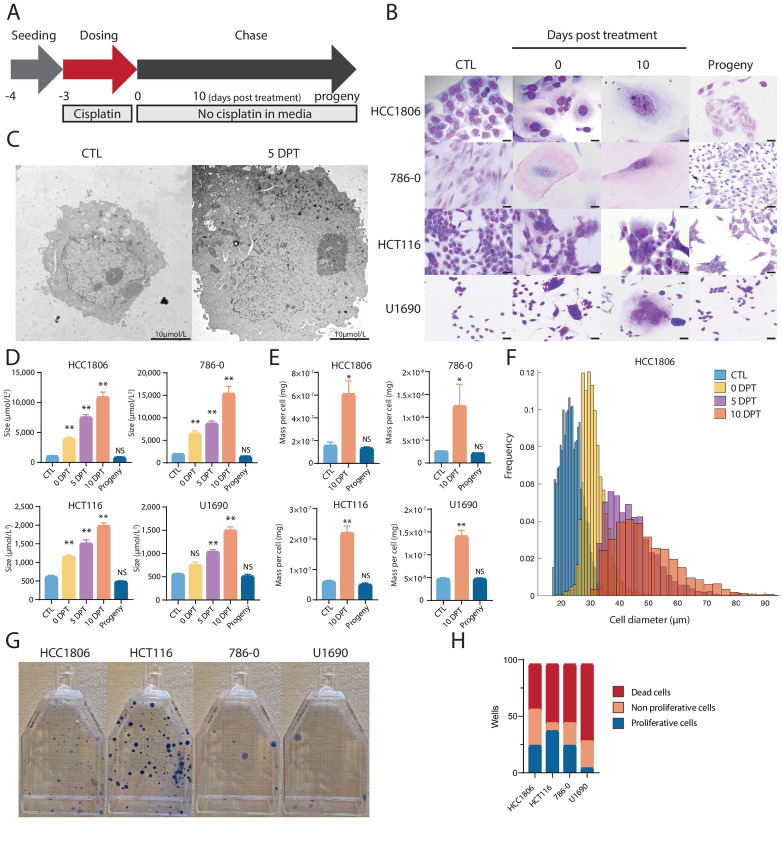
Drug-resilient cells triple in size and mass for up to 10 days posttreatment. **A,** Our treatment protocol entailed that seeded cells were treated with cisplatin (T = −3 days) for 72 hours (T = 0 days posttreatment; DPT) and, following filtration, studied for 10 DPT. After a subsequent time interval (between 2–12 weeks depending on cell line), surviving cells gave rise to progeny. **B,** Cells from four cancer cell lines stained with Giemsa when untreated (CTL) and treated at timepoint 0 DPT, 5 DPT, and 10 DPT, and progeny from these cells at 28 DPT (HCC1806, 786-0, and HCT116 cells), and 49 DPT (U1890 cells; *n* = 3 biological replicates). Scale bar, 20 µm. **C,** Detailed view of nuclei of untreated HCC1806 cells and when surviving 5 DPT, using TEM. Scale bar, 2 µm. **D,** Size of untreated (CTL) cells, surviving cells at 0 DPT, 5 DPT, 10 DPT, and progeny at 28 DTP (HCC1806, 786-0), 21 DTP (HCT116), and 49 DTP (U1890). Sizes acquired by imaging of adherent cells and analyzed in ImageJ. Cell size average from biological triplicates (*n* = 3) and *P*-value (^∗∗^, *P* < 0.01; ^∗^, *P* < 0.05; NS, not significant) by ANOVA test as indicated. **E,** Mass of untreated (CTL) cells, surviving cells at 10 DPT, and progeny at 28 DTP (HCC1806, 786-0), 21 DTP (HCT116), and 49 DTP (U1890). Cell mass average from biological triplicates (*n* = 3) and *P*-value (^∗∗^, *P* < 0.01; ^∗^, *P* < 0.05; NS, not significant) by ANOVA test as indicated. **F,** Cell diameter distributions and frequency of 10,000 sorted HCC1806 cells in control (CTL) and treated cells at 0 DPT, 5 DPT, and 10 DPT (biological replicates *n* = 3). **G,** Representative image of proliferating clones of progeny 28 DTP (HCC1806, 786-0, HCT116, and U1890). Cells are stained with 0.5% crystal violet solution. **H,** Distribution of surviving cells that died or regained proliferative capacity 2 months after treatment of HCC1806, 786-0, HCT116, and U1890 cells. Treated and filtered cells (*n* = 96) at 0 DPT were transferred to individual wells. Average of the number of wells with dead cells, large cells, and proliferating progeny cells from biological replicates (*n* = 3) and *P* value (^∗∗^, *P* < 0.01; ^∗^, *P* < 0.05, significant relative to vehicle) by ANOVA test as indicated.

Cells were seeded in 10 mm dishes (5 × 10^5^ cells per dish) overnight and dosed with cisplatin at their respective LD_50_ for 72 hours. Cells were then trypsinized, size filtered (using 40 µm mesh filter; Nordic Diagnostica, PS-43-50040-03), and reseeded or analyzed (Supplementary Data S1). The reseeding timepoint at 72 hours was set as the day 0 timepoint (Fig. 1A). Reseeded cells were maintained in culture until colonies started to form. The LD_50_ was estimated at the 72 hours timepoint. When monitored for 10 more days, 1%–10% of the reseeded cells consistently survived. At the day 10 timepoint, all surviving cells displayed a large phenotype (>3-fold larger than untreated cells) and were nondividing.

### Generation of CRISPR/Cas9 KO Cell Lines

Cells were transduced with a doxycycline-inducible Cas9 lentiviral plasmid (Horizon Bioscience, #VCAS11227). Cas9 was induced by treatment with 1 µg/mL doxycycline for 24 hours before electroporation using Amaxa HT nucleofector following the manufacturer's instructions (4 × 10^5^ cells, Amaxa SF Cell Line 4D-Nucleofector Kit S, #V4SC-2096, program EN-130-AA) for single-guide RNA uptake. Post-electroporation viable cells were expanded and electroporation (Lonza, #V4XC-9064) was repeated on pools of cells for a total of three times. KOs were validated via DNA sequencing and Western blotting. For guide sequences, see Supplementary Table S2.

### Giemsa Staining

A total of 1 × 10^5^ cells were seeded in 6-well plates with a coverslip at the bottom of each well. Cells were left to attach overnight and then treated with cisplatin at the respective LD_50_ concentration. After 72 hours, surviving cells were collected at 0, 5, and 10 days. Wells were washed with PBS and 1 mL of methanol:acetone (1:1), after which the plates were frozen overnight at −20°C. A total of 1 mL/well Giemsa (Merck, #48900) was added and for a following 1-hour incubation, the wells were washed three times with PBS. Coverslips were then mounted and imaged using slide scanner (Olympus).

### Transmission Electron Microscopy

Cells were trypsinized, washed, and fixed in 4% paraformaldehyde and 4% glutaraldehyde in 0.1 mol/L Sorensen phosphate buffer for 2 hours. The cells were then post-fixed in 1% osmium tetroxide and embedded in low melting agarose. Dehydration was carried out with increasing concentrations of acetone and the cells were then embedded in Polybed 812. Samples were sectioned with Ultratome Leica EM UC7 with a Diatom diamond knife at 60 nm thickness onto Pioloform-coated Maxtaform H5 copper grids. Samples were analyzed using a Tecnai 120 kV microscope (at 100 kV) and imaged with a Veleta camera.

### Quantification of Surviving Cell Numbers, Size, and Weight

Surviving cells were generated as described above, trypsinized and suspended in 50 mL of DMEM. Cell sizes of HCT1806, HCT116, and 786-0 were quantified after treatment at the 0 DPT, 5 DPT, and 10 DPT and when untreated control (CTL) by imaging 10,000 cells using a high throughput particle analyzer (“FlowCam”: Yokogawa Fluid Imaging Technologies, Inc.). Measures of cell sizes were acquired from the FlowCam output. A Gaussian mixture model was used to identify and quantify distinct cell populations classified by diameter (Supplementary Data S1). For most timepoints, two populations were identified, with one population having a substantially larger diameter than the other. In most cases, the population with the smaller diameter was the most frequent. In some instances, three populations were identified, as the two populations’ model was not enough to recover the observed size distribution. The code identifying the cell population using the Gaussian mixture model was written in MATLAB (Supplementary Data S1).

The Kolmogorov–Smirnoff test was used to compare the experimental distribution against the normal hypothesis. To explore whether the sample could come from a truncated normal distribution, we used the “mle” function of MATLAB with the option “TruncationBounds”. The “mle” function was also used for the fit to a Gaussian mixture model, with the option “pdf” to fit to a custom distribution. This custom distribution was defined as a convex combination of a normal distribution, with either two terms for the two components model or three terms for three components model. The Kolmogorov–Smirnoff test was then used to depict whether the sample could be generated by the fitted theoretical distributions.

To quantify the mass of the cells, tin cups (IVA analysentechnik GMBH) were weighed individually prior to experimentation and kept in a 96-well plate. Cells were trypsinized, counted, and resuspended into 1 mL of PBS (roughly 20 million control cells, and 2 million surviving cells). Cells were centrifuged and resuspended into 100 µL PBS and transferred into a tin cup. Tin cups were kept open (under a lid in the 96-well plate) and frozen at −80°C. The samples were subsequently freeze dried (Lyph-Lock 12 freeze dryer, Labconco). Afterward, each tin cup was weighed and differences in weights were calculated for each condition in biological triplicates.

### Immunoblotting

Cells were washed with PBS and lysed in 8 mol/L urea lysis buffer (8 mol/L urea, 20% SDS, 100 µL/mL glycerol, 1.5 mol/L TRIS pH 6.8) with protease (Merck, #P8340) and phosphatase inhibitor cocktails (Merck, #P5726). Cell lysates separated by 10% SDS-PAGE at 300 V for 15 minutes (Bio-Rad, #4561094) were transferred to nitrocellulose membranes (Bio-Rad, #1704270). The membranes were blocked for 5 minutes using EveryBlot Blocking Buffer (Bio-Rad, #12010020), incubated with primary antibodies for 1 hour, washed for 30 minutes with Tris-buffered saline Tween 20 (TBST) and incubated with fluorescent secondary antibodies to probe for multiple targets on each membrane for 1 hour, washed for 30 minutes and imaged using Bio-Rad Chemidoc (Bio-Rad). Antibodies are denoted in Supplementary Table S3.

### scWGS

For scWGS, surviving cells were size filtered and individual nuclei were manually placed into wells and control cells sorted by a BD FacsJAZZ cell sorter (BD Biosciences). For single-nuclei isolation, cell pellets were resuspended in lysis buffer [1 mol/L tris-HCl pH 7.4, 5 mol/L NaCl, 1 mol/L CaCl_2_, 1 mol/L MgCl_2_, 7.5% BSA, 10% NP-40, ultra-pure water, 10 mg/mL Hoechst 33358, 2 mg/mL propidium iodide (PI)] and kept on ice in the dark for 15 minutes to facilitate lysis. Single nuclei, as assessed by PI and Hoechst staining were sorted into 96-well plates and stored at −80°C until further analysis. For library preparation, single nuclei were lysed and DNA was barcoded, followed by automated library preparation (Bravo Automated Liquid Handling Platform, Agilent Technologies) as described previously (27). Single-cell libraries were pooled and analyzed on an Illumina Hiseq2500 sequencer (Illumina). Sequencing was performed using NextSeq 500 machine (Illumina; up to 77 cycles; single end) Full analysis methods can be found in Supplementary Data S1. The bioinformatics analysis to calculate the read-depth ratio used the software BWA (0.7.17) for alignment of sequence reads to the reference genome (hg19); Samtools (1.17; ref. 28) was used for filtering and sorting the aligned reads; GATK (4.0.8.1), Bcftools (1.17; ref. 28), and Eagle (2.4.1; ref. 29) were used for variant calling, filtering, and variant phasing, respectively; and finally Chisel (1.1.4; ref. 30) was used for read-depth calculations and plotting. Analysis of copy-number change was performed using AneuFinder (3.17; ref. 31). Full analysis methods can be found in Supplementary Data S1.

### RNA-seq

RNA was extracted using TRIzol and was subsequently DNAse digested using DNase I from RNAqueous Micro Kit (Invitrogen, #AM1931) with RNase inhibitors (Invitrogen, #10777-019) with merged protocol of (#10777-019). Quantification of mRNA levels was undertaken using Qubit and RNA integrity number (RIN) values generated using BioAnalyser. Library preparation, bulk sequencing, and data analysis were performed by Novogene (full methods in Supplementary Data S1). In brief, 1 µg RNA per sample was used as input material for RNA preparations. Sequencing libraries were generated using NEBNext Ultra RNA Library Prep Kit for Illumina (NEB) following the manufacturer's recommendations. Library preparations were sequenced on an Illumina platform and paired-end reads were generated. Transcription factor analysis was done as described previously (32).

### ATAC-seq

Cells were washed twice with media prior to DNase I (Stem cell Technologies, #07900) treatment. 100x DNase solution (20,000 UN/mL) and 100x buffer (250 mmol/L MgCl_2_ and 50 mmol/L CaCl_2_ in dH_2_O) were added to tissue culture media and the cells were incubated at 37°C for 30 minutes. Cells were subsequently washed, trypsinized, and counted. A total of 100,000 cells per replicate were cryopreserved in a solution with 50% FBS, 40% growth media, and 10% DMSO at −80°C degrees. Library preparation, sequencing, and bioinformatics analysis were performed by Activemotif. Full methods and analysis pipeline can be found in Supplementary Data S1.

### FISH

FISH was carried out according to standard methods using centromere-specific or locus-specific probes (Vysis CEP X (DXZ1) SpectrumGreen Probe, Vysis CEP 1 SpectrumOrange Probe, Vysis CEP 2 (D2Z1) SpectrumOrange Probe, Vysis LSI 19q13 SpectrumOrange/19p13 SpectrumGreen Probes, Abbott Scandinavia). For interphase FISH, a minimum of 200 nuclei were analyzed for each probe.

### Statistical Analysis

Data were compared with the normal distribution using the Shapiro–Wilk test in the GraphPad prism software (version 9.5.1). One-way ANOVA was used to determine statistical significance for Western blot samples. For cell mass and inhibition studies, the Kruskal–Wallis test was used to determine significance. Cell size data were acquired from acquired from >2,500 cells obtained using FlowCam images of >2,500 cells (Yokogawa Fluid Imaging Technologies, Inc.). The cell size populations were separated and quantified with the Gaussian mixture model with two components that was able to fit all the experimental distributions statistically analyzed.

### Data Availability

Raw data are available at Gene Expression Omnibus under accession number GSE235909 and at SRA under accession number PRJNA990979. All scripts containing the exact commands used for the analysis of scWGS are publicly available on GitHub (https://github.com/aboffelli/pacc-copy-number). All other data are available from the corresponding author upon reasonable request.

## Results

### Cancer Cells Survive in Response to Cytotoxic Drugs by Increasing in Size

To investigate the phenotype of therapy-resilient cancer cells, we treated different cancer cells with cisplatin. Cancer cell lines derived from breast (HCC1806), colon (HCT116), lung (U1690), and kidney (786-0) carcinomas were treated with cisplatin at different concentrations (2–10 µm). The respective LD_50_ was calculated after 72 hours following treatment (Supplementary Fig. S1; Supplementary Table S1). After treatment, we allowed the cells to recuperate (Fig. 1A). The surviving cells in all four cell lines at 10 DPT demonstrated a significant increase in nuclear and cell size (Fig. 1B). This phenotype was also noted in six additional cancer cell lines (Supplementary Fig. S2). An increase in nuclear size in surviving HCC1806 cells at 5 DPT was identified using transmission electron microscopy (TEM; Fig. 1C). In Supplementary Fig. S3, there is a representative image showing the increase in nuclear size and an increase in structures likely to be peroxisomes or lipid droplets to perform oxidative reactions (33). Average cellular sizes (untreated, treated, and subsequent daughter cells of the treated cells, i.e., progeny) were measured with two-dimensional imaging of adherent cells, which identified an increase in cell size of all cancer cell lines as compared with 0 DPT (3- to 5-fold) that continued to 10 DPT (9- to 11-fold, Fig. 1D). This quantification demonstrated that progeny cells were of similar size to untreated control cells (Fig. 1D). Cellular mass increased on average 2.8 times between 0 and 10 DPT (Fig. 1E). Cell size measured with the FlowCam showed an average increase in three cell lines of 1.4 times at 0 DPT, 2.0 times at 5 DPT, and 2.3 times at 10 DPT (Fig. 1F; Supplementary Figs. S4–S7).

### Surviving Large Cells have the Capability to Produce Progeny

The surviving treated cells remained large and nonproliferative for a period of 2 to 8 weeks before returning to a proliferative state. The characterization of this nonproliferative period is beyond the scope of this work but shares aspects with senescence-like cell state. Their resulting progeny had a cell size and mass like those of untreated control cells (Fig. 1D–F). To determine the efficiency at which progeny were produced, clonogenic assays were performed and cells were stained 4 weeks after the seeding of surviving cells. We observed that all four cell lines had produced colonies 4 weeks posttreatment (Fig. 1G). To determine the rate at which treated and surviving cells could generate progeny, we transferred treated single cells that had been size filtered using a 40 µm filter to individual wells in a 96-well plate. The number of nonproliferative cells (larger size), proliferative cells (smaller size, i.e., colonies of progeny), and dead cells were measured. Cells were dead in 41%–78% of the wells, while large singular nonproliferative surviving cells remained in 7%–41% of the wells, and proliferating colonies were observed in 6%–35% (one plate per cell line, Fig. 1H; Supplementary Table S4). These data suggest that large cells can eventually divide and produce viable progeny which continue to proliferate.

### Large Surviving Cancer Cells Undergo Whole-genome Duplication

**FIGURE 2:**
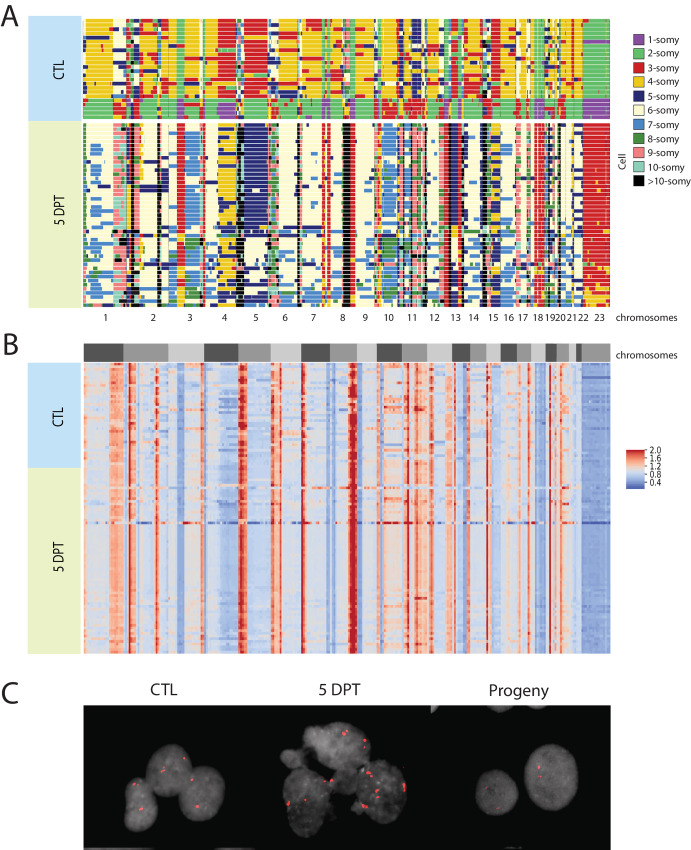
Drug-resilient cells exhibit one to two whole-genome duplications with high fidelity. **A,** Copy numbers in untreated and treated surviving HCC1806 cells 5 DPT, as visualized with AneuFinder (reads per 10 Mb over total amount of reads) from scWGS each row representing a single nucleus. **B,** Ratio of DNA content within each cell in untreated and surviving HCC1806 cells 5 DPT. The heat maps show the normalized read depth (reads per 10 Mb bins over total amount of reads in the cell) of scWGS, where blue areas show a lower number of reads, and red areas show a higher number of reads. The blocks R1, R2, and R3 in the left represent replicates 1, 2, and 3, respectively. **C,** Copy number of chromosome X in untreated (CTL), surviving HCC1806 cells at 5 DPT and their progeny, as visualized with chromosomal FISH of cells in interphase.

To determine therapy-induced genetic changes, we performed scWGS of the breast cancer cells (HCC1806), untreated control cells and surviving cells (Fig. 2A). To this end, untreated single control cells were sorted into 96-well plates using flow cytometry. Because the size of nuclei in the surviving cells hampered FACS, individual cells at 5 DPT were manually transferred to 96-well plates. Control cells were selected for sequencing from the main peak based on Hoechst/PI staining and FACS. We found that in most control cells, chromosomes were disomic (2, 5, 6, 12, 13, 14, 21), trisomic (1, 3, 4, 7, 8, 9, 16, 17, 20, 22), or monosomic (10, 13, 15, 18, X). In surviving cells, most chromosomes were duplicated several times (with the cells containing multiple copies of each chromosome) and showed a higher copy-number compared with the control cells (Fig. 2A). After duplication, the proportion of DNA in each chromosome continued to be the same, as demonstrated by a calculated read-depth ratio for the HCC1806 cells (Fig. 2B). The same trend is visible for the 786-0 cells (Supplementary Fig. S8). That the proportion of DNA remained intact indicated that the whole genome was doubled, keeping the fidelity of the original rearrangements in the control cells. The high-fidelity duplication event would suggest that the surviving cells were independent on any exact chromosomal karyotype bias. Moreover, we quantified the karyotype heterogeneity between individual cells on each chromosome in each condition to describe overall heterogeneity score. It revealed a lower heterogeneity between the surviving HCC1806 cells compared with the heterogeneity within the untreated control cells (Supplementary Table S5, with the reverse trend for the 786-0 cells). We validated the scWGS ploidy assessment of surviving cells during the transient polyploid state using interphase FISH (iFISH) with centromere probes for chromosomes 1, 2, and X, and a locus specific chromosome 19 probe (Supplementary Table S6). Centromere probes confirmed an increased copy number of chromosome X (as a validation of the fold changes observed in the WGS) in the surviving HCC1806 cells with the CTL cells containing two copies due to the cells being in G_2_ state (Fig. 2C). The same trend is visible for the 786-0 cells (Supplementary Fig. S8). Therefore, the surviving cells had undergone at least one high fidelity whole genome duplication by 5 DPT while not having divided.

### Chromatin Regulation Emerges in Large Surviving Cells

**FIGURE 3:**
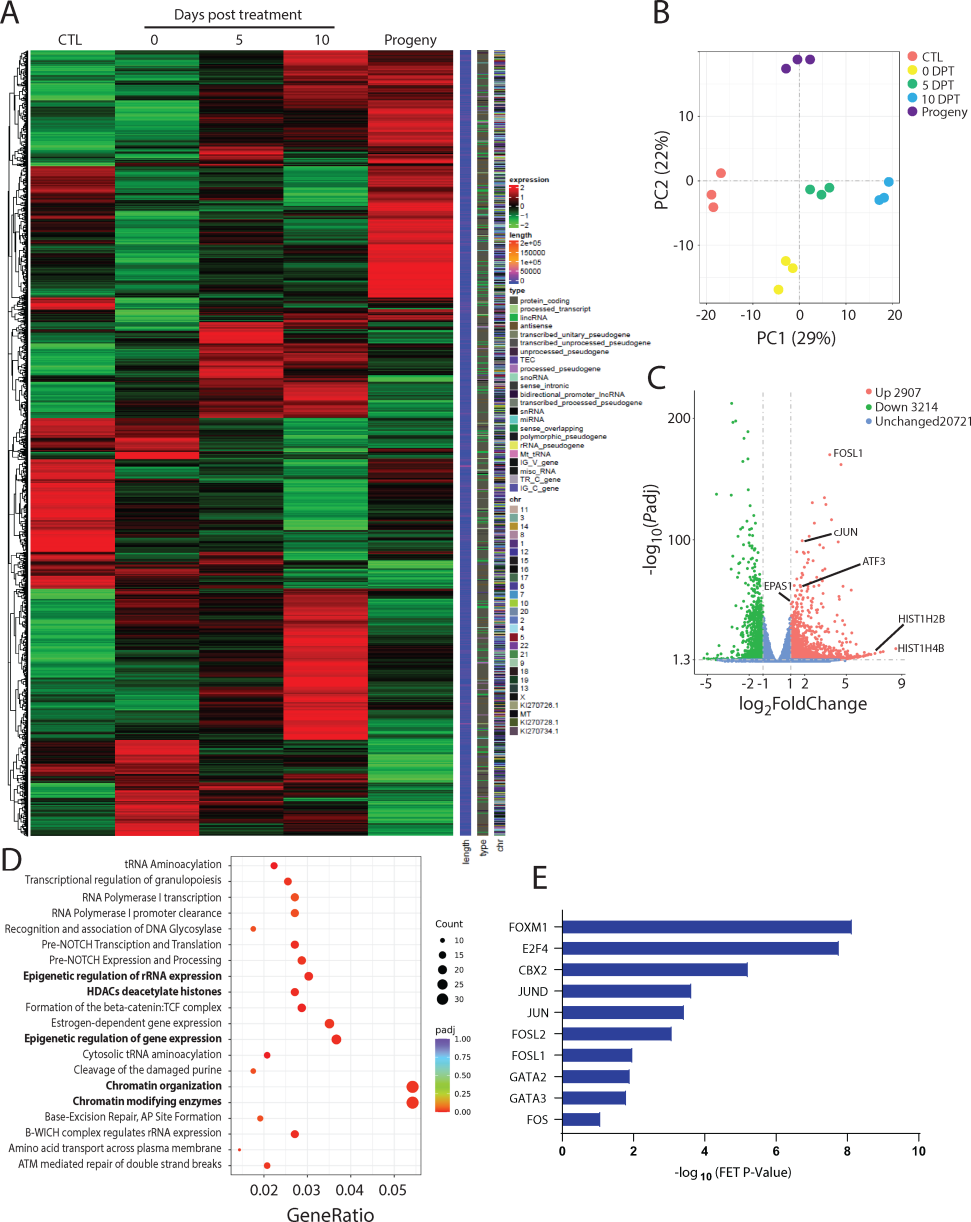
Cisplatin treatment of HCC1806 cells induced an altered transcriptome. **A,** Visualization of clusters of genetically similar cell populations (HCC1806) when untreated (red), and when resilient to treatment and large at 0 DPT (yellow), 5 DPT (green), and 10 DPT (blue). The PCA based on differentially expressed genes from the RNA-seq data. **B,** Visualization of gene expression of HCC1806 cells when untreated, surviving treatment at 0 DPT, 5 DPT, 10 DPT, and as progeny. Heat maps of the differentially expressed gene data. **C,** Visualization of downregulated and upregulated genes (fold change vs. adjusted *P*-value) in drug-resilient and transiently large HCC1806 cells at 10 DPT, compared with untreated control cells. **D,** Pathways upregulated in HCC1806 cells surviving at 10 DPT, as quantified with RNA-seq and Reactome analysis. **E,** Transcription factors regulating upregulated genes in HCC1806 cells surviving 10 DPT as quantified using RNA-seq and CHEA3 analysis.

To investigate changes in the transcriptome, we performed RNA-seq. In HCC1806 cells, changes in transcriptional expression were noticed immediately after exposure to cytotoxic treatment, and during the transiently large state. There were clusters of transcriptional expression changes that were distinct between untreated cells and surviving cells (e.g., 10 DPT), between the surviving cells of different ages (0 to 10 DPT), between the surviving cells at 10 DPT and progeny cells, and between untreated cells and progeny cells (Fig. 3A). Principal component analysis (PCA) demonstrated the following differences in comparison with untreated cells: large surviving cells at 0 DPT were the most different along PC2 (representing 22% of the differentially expressed genes in the dataset), large surviving cells at 10 DPT were the most different along PC1 (representing 29% of differentially expressed genes in the dataset), and progeny cells were the most different along PC1 (Fig. 3B). A total of 2,907 genes were upregulated in HCC1806 cells at 10 DPT compared with untreated control cells, including *EPAS1*, *FOSL1*, and the histone genes *H2BE* and *H4BE* (Fig. 3C). A total of 3,214 genes were downregulated in HCC1806 cells at 10 DPT, including *BPIFB1*, *PAX7*, and *CDH5* (Fig. 3C). Many upregulated pathways between untreated and treated large surviving cells at 10 DPT related to, for example, chromatin regulation (Fig. 3D). Downregulated pathways between untreated and treated, large, surviving cells at 10 DPT relate to, for example, glycosylation, retinoic acid signaling, and non-integrin membrane-ECM interactions (Supplementary Fig. S9). Analysis of transcription factors involved in the regulation of the differentially upregulated genes in surviving cells at 10 DPT were members of the JUN, *FOS*, *FOXM1*, *E2F4*, *CBX2*, and *GATA* families (Fig. 3E). Transcription factors involved in the downregulated genes were *FOXA1*, *ESR1*, and *RFX5* (Supplementary Fig. S10). Therefore, large transcriptional rewiring appears necessary for posttreatment cell survival, with many of these changes affecting histones and stress response. We then moved on to explore to what effect this would have on protein expression.

### Proteins of the Minichromosome Maintenance Complex is Reduced in Surviving Cells

**FIGURE 4:**
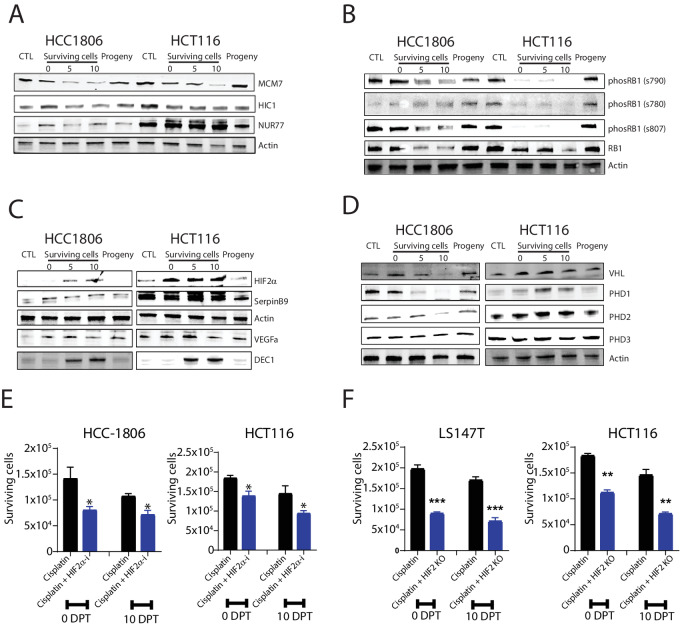
Protein changes validate the role of HIF2α and RB1 for cell survival. **A,** Protein level changes of HIF2α-interacting proteins, MCM7, HIC7, and NUR77 in HCC1806 and HCT116 cells when untreated (CTL), when surviving at 0 DPT, 5 DPT, and 10 DPT and as progeny; demonstrated by Western blot analysis. Actin was used as a loading control. Molecular weight markers in kDa are shown to the left. **B,** Representative images of protein level changes of RB1 and its phosphorylated sites (s790, s780, and s807) in HCC1806 and HCT116 cells when untreated (CTL), when surviving at 0 DPT, 5 DPT, 10 DPT and as progeny; as determined by Western blot analysis. **C,** Protein level changes of HIF2α and its targets SERPINB9, VEGF, and DEC1 in HCC1806 and HCT116 cells when untreated (CTL), as surviving at 0 DPT, 5 DPT, and 10 DPT and as progeny; as determined with Western blot analysis. **D,** Protein level changes of VHL and PHD1–3 in HCC1806 and HCT116 cells when untreated (CTL), when surviving at 0 DPT, 5 DPT, and 10 DPT, and as progeny; as determined with Western blot analysis. **E,** Number of HCC1806 and HCT116 cells surviving at 0 DPT and 10 DPT when treated with cisplatin only or cisplatin together with the HIF2α inhibitor Belzutifan. **F,** Number of LS174T and HCT116 colon cancer cells surviving cisplatin at 0 DPT and 10 DPT as “normal” and with k HIF2α KO from biological replicates (*n* = 3) and *P*-value (^∗∗^, *P* < 0.01; ^∗^, *P* < 0.05; significant relative to vehicle) by ANOVA test as indicated.

Epigenetically regulated gene expression and maintenance of chromosomal stability requires the interaction of many proteins in a regulated manner through the cell cycle. For example, the expression of the minichromosome maintenance complex (MCM) proteins regulates the initiation of genome replication via its formation of the prereplication complex. Expression of *MCM7*, which was highly upregulated in the RNA-seq data, was reduced in surviving cells in a time-dependent manner, indicating a slowing of genome replication as cellular size increased to the maximum (Fig. 4A). In contrast, the chromosomal stabilizing HIC1 protein that interacts with cyclin D1 was relatively unaffected in the surviving cell state. Moreover, NUR77, a hypoxia-inducible protein which can bind to AP-1 promoters and mediates both cell cycle progression and apoptosis, was upregulated in surviving cells. The expression of these proteins indicates that, rather than the cell cycle checkpoint blockade, the replication of DNA may be limiting growth of surviving cells. However, as the cell cycle was clearly altered with surviving cells not dividing, we decided to further investigate cell cycle perturbations via the RB1 protein.

### RB1 Expression is Downregulated in Surviving Cells

The growth and whole-genome doubling of surviving cells suggest that cells undergo repeated S-phases without mitosis, which requires that checkpoints are skipped. A major cell cycle (G_1_–S and S) checkpoint regulator is the RB1 (34), which also has chromatin remodeling functions (34). Expression of total RB1 was reduced in a time-dependent manner but returned to baseline levels in proliferative progeny (Fig. 4B). Phosphorylation of RB1 results in cell cycle progression by preventing RB1 to bind to E2F transcription factors that alters the transcription of genes that facilitate G_1_ progression (35). In surviving treated cells, the phosphorylation of Ser790 and Ser807 followed the same pattern as total RB1 expression, whereas phosphorylation of Ser780 was absent in surviving treated cells (Fig. 4B). In combination with the data demonstrating cell cycle progression, the reduction in RB1 thus indicates that surviving cells transition through the G_1_–S checkpoint.

### Inhibition of HIF2α Reduce the Number of Surviving Cells

*EPAS1* (encoding HIF2α) was upregulated in surviving cells across different timepoints, cell types, and treatments (Supplementary Fig. S11). Stabilization of HIF2α is described to canonically occur under hypoxic conditions. However, similar to what was observed here, increasing evidence suggest that HIF2α can be stabilized under physiologic oxygen conditions (5%–7% O_2_) in a tissue and time-specific manner (36–38). Stabilization of HIF2α and activation of downstream signaling is known to result in significant transcriptional changes in cells including an altered cell cycle (39). After chemotherapy treatment, we found that HIF2α was stabilized at the protein level in both cell lines (Fig. 4C) Thus, we focused on its downstream targets.

Expression of the HIF2α target *SERPINB9* increased in surviving cells as well as in progeny populations, while *DEC1* was expressed only at timepoints 5 DPT and 10 DPT (Fig. 4C). The *VEGFa* was undetected in control cells but was expressed in the polyploid surviving cells and their progeny (the expression peaked at 5 DPT; Fig. 4C). We then asked whether HIF2α stabilization in surviving cells is coupled to the Von Hippel Lindau protein (VHL) and Prolyl hydroxylase (PHD) activity. We measured HIF1α activity as a proxy because this protein is stabilized in the absence of VHL. We did not detect HIF1α or the canonical downstream target *CAIX* in survivor cells, and PHD3 expression was unchanged (Fig. 4D). While PHD1 was downregulated in HCC1806 and upregulated in HCT116 cells, the reverse occurred for PHD2. Expression of VHL was increased following treatment (Fig. 4D). These observations suggest that VHL and PHD activities are uncoupled to HIF2α stabilization in surviving HCT116 cells and that other noncanonical mechanisms are involved in facilitating HIF2α signaling. In the case that HIF2α stabilization independently contributes to cell survival, we asked whether inhibition of HIF2α (via inhibiting the formation of the HIF2α-HIF1β heterodimer required for transcription activation) reduced cell survival, which indeed was the case (Fig. 4E). Moreover, we tested the effect of Notch inhibition with a reduction in survival by at least 20% by 10 DPT (Supplementary Fig. S12). Examining the effect of HIF2α on cell survival using previously validated *EPAS1* CRISPR/Cas9-KO cell lines (HCT116 HIF2-KO and LS174T HIF2-KO cells, because we were unsuccessful in generating *EPAS1* KOs in HCC1806 cells), we found that survival was reduced by >50% in *EPAS1* KO cells at timepoint 10 DPT (Fig. 4F). In conclusion, signaling via AP-1 and HIF2α are at least in part important for survival of cisplatin therapy via the transient formation of a large cell state.

### The Chromatin Landscape is Remodeled in Surviving Cells

**FIGURE 5:**
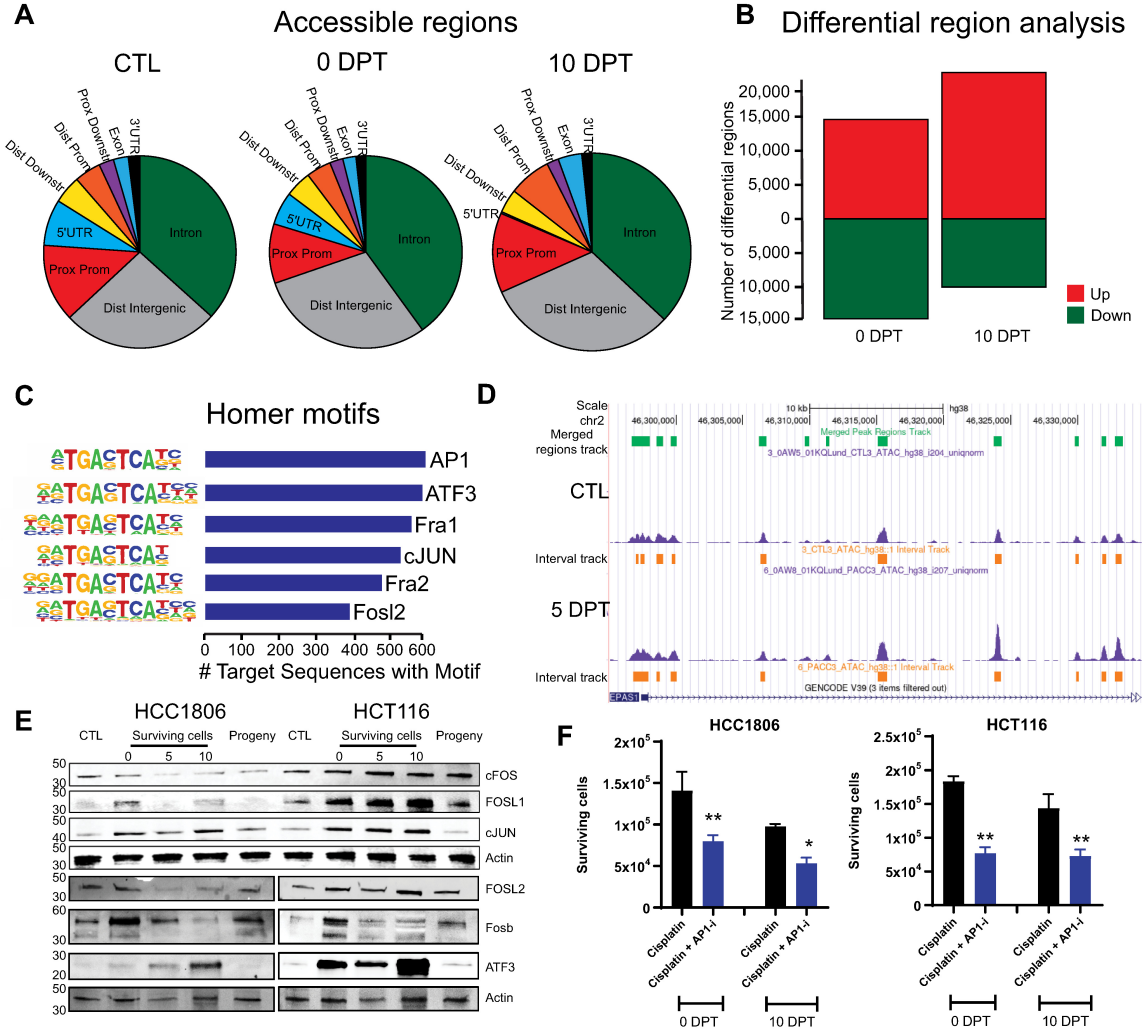
Surviving polyploid cells demonstrate an overall reduction of chromatin openness while AP-1 motifs were enriched. **A,** Visualization of accessible regions in surviving HCC1806 cells at 0 DPT; as quantified by ATAC-seq. **B,** Visualization of more (green) or less (red) accessible regions in surviving HCC1806 cells at 0 DPT; as quantified by ATAC-seq. **C,** Visualization of DNA motifs for AP-1 family members in HCC1806 surviving at 0 DPT, as quantified by ATAC-seq. **D,** Openness of region for *EPAS1* in HCC1806 cells surviving at 0 DPT, as visualized with genome browser tracks. **E,** Protein level changes in HCC1806 and HCT116 cells of the AP-1 members FOS, FOSL1, JUN, and ATF-3 in untreated (CTL), surviving cells at 0 DPT, 5 DPT, and 10 DPT, and as progeny. **F,** Number of HCC1806 and HCT116 cells surviving at 0 DPT and 10 DPT when treated with cisplatin alone and cisplatin together with the FOS/AP-1 inhibitor T-5224 from biological replicates (*n* = 3) and *P*-value (^∗∗^, *P* < 0.01; ^∗^, *P* < 0.05, significant relative to vehicle) by ANOVA test as indicated.

Because epigenetic-modifying proteins consistently displayed increased expression in surviving cells across cell types and timepoints, we investigated the chromatin landscape using ATAC-seq in the breast and colon cancer cell lines. Surviving HCC1806 cells had a higher proportion of open distal intergenic regions and of intron regions, but a smaller fraction of open proximal promoters and 5′-UTR (untranslated regions; Fig. 5A). Differential region analysis showed that chromatin, in general, was less accessible in surviving cells compared with untreated cells at 0 DPT. However, by 10 DPT chromatin was more accessible compared with untreated control cells (Fig. 5B). Enrichment analysis of promoters that were more open in the surviving cells identified a high frequency of AP-1 binding sites, in particular the promoter regions downstream of the target genes *FOSL1*, *FOSL2*, and *JUN* (Fig. 5C). However, other downstream genes with AP-1 motifs were among downregulated hits (e.g., *JunB*), suggesting that other co-regulating factors besides AP-1 are involved for cells to survive through a transient state of polyploidy. We did not note any changes in the chromatin landscape around *AP1* gene members themselves. The chromatin landscape surrounding the *EPAS1* gene was more open in surviving treated cells than in untreated cells (Fig. 5D). This suggests that increased transcription is a possible mechanism by which *EPAS1* expression is increased as opposed to posttranslational mechanisms alone and that HIF2α is important in mediating survival.

### Targeting AP-1 Subunits in Surviving Cells Decrease Survival

To assess whether AP-1 subunits were also translated into protein at higher level rather than just transcribed in surviving cells, we determined the expression of AP-1–regulated proteins (FOS, JUN, and FOSL1) in HCC1806 and HCT116 cells, because these lines produced the highest fraction of proliferating cells after cisplatin treatment (Fig. 1H). Expression of FOS was decreased in HCC1806 but increased in HCT116 cells following treatment cessation (Fig. 5E). In HCC1806 cells, FOSL1 was only expressed immediately following treatment cessation and in surviving cells 10 DPT. In contrast, FOSL1 was increased in HCT116 cells following treatment and returned to baseline levels in progeny. Expression of JUN was increased in surviving cells in both cell lines suggesting a possible targetable subunit across cancers (Fig. 5E).

To determine the relevance of the findings that AP-1 signaling is important for survival, we combined cisplatin treatment with AP-1 inhibition using T2445 (which specifically inhibits the FOS/JUN heterodimer). We saw no effect on cellular proliferation of T2445 on its own (Supplementary Fig. S13). We quantified the number of surviving cells at timepoints 0 DPT and 10 DPT after the combined treatment with cisplatin for 72 hours. Our data showed that inhibition of AP-1 reduced survival by ≥50%, at both timepoints (Fig. 5F) thus showing that AP-1 signaling via cFOS/cJUN heterodimer activity plays a role in the formation of surviving cells.

## Discussion

Resistance to systemic therapies is commonly thought to be due to tumor heterogeneity and acquired mutations that are further fueled by aneuploidy, genetic instability, or both. However, cells can also survive stress through transient and phenotypic changes, including cell size. In other organisms (e.g., protists, plants, and prokaryotes), these transient changes in cell size via cell-autonomous whole-genome doubling are an adaptive response to environmental stress (11). In this study, we found that cancer cells circumvent therapy-induced death through a state of repeated whole-genome doubling resulting in transient polyaneuploidy. These data indicate that reversible alterations to the cell cycle allow cells to survive cytotoxic treatment. We further demonstrated that the entry into the transiently morphologically large and drug-resilient state induced cellular stress responses.

Alterations to the canonical mitotic cell cycle were found in a recent study of drug-resilient, large, and primarily mononucleated prostate carcinoma cells (40). In that study, Kim and colleagues (2023) demonstrated that upon exposure to cytotoxic drugs, cells continue to replicate DNA by exiting the proliferative mitotic cycle and entering an endocycle (40). In another study of p53-mutated lymphoma cells, the cells after treatment failed to arrest in G_1_ but instead at G_2_ before entering an endocycle, while functional p53 stopped this (22). In the alternative cell endocycle, cells skip mitosis and progress through multiple rounds of G- and S-phases that result in cellular hypertrophy and repeated whole-genome doublings. The repeated DNA synthesis (S-phase) without cell division in the surviving cells of this study would also be consistent with an endocycle proceeding through multiple cell cycle checkpoints and avoids checkpoint-mediated apoptosis. Cancer cells undergoing polyploidy appear to limited to 32 copies of a chromosome (32C or 4 endocycles), which aligned with our results by interphase-FISH (22). By tracking the changes in transcriptional expression of the large cells that survive cytotoxic chemotherapy, we showed that cell cycle regulators AP-1 and RB1, as well as stress-responsive HIF2α were altered in the entry into the adaptive prosurvival state. We hypothesized that these altered pathways represent a stress-induced response leading to an active cell cycle across checkpoints that confers protection from cytotoxic agents acting on proliferative cells.

Our RNA-seq data indicated that the AP-1 pathway is altered in breast and colon cancer cells that survive chemotherapy treatment and adopt a large cell size. The AP-1 transcription factors are activated in response to stress, regulate processes such as proliferation and apoptosis (41), and play a key role at the G_1_–S transition point (42). In addition to direct phosphorylation and dephosphorylation of AP-1 subunits, AP-1 activation is influenced by transcriptional regulation of its dimer members ATF, FOS, or JUN. We found that the ATF-3 protein accumulates as transiently large cells form in HCC1806 and HCT116 cell lines following treatment. Depending on baseline expression levels, ATF-3 has been implicated in both the promotion and inhibition of proliferation (43, 44). Dysregulation of the FOS and JUN family is associated with cancer therapy resistance and poor patient survival (45–47). For example, loss of FOS indicates worse overall survival in patients with breast cancer (45) while increased expression of FOSL1 and JUN family members promotes drug resistance and growth in breast and colorectal cancer cells (46, 47). Although our findings are consistent with AP-1 being involved in stress responses and cell cycle alterations that mediate drug resilience, inhibition of AP-1 did not entirely abrogate cell survival by the state of polyploidy. While it is possible that this is due to suboptimal specificity of the inhibitor itself, it may also indicate that other mechanisms conjoin to allow the altered cell cycle.

Cell cycle progression into S-phase can be mediated by the inactivation of RB1, which occurs either by phosphorylation, genetic deletion or mutation, chromatin-modifying enzymes or by binding to viral oncoproteins (34). We found that the total RB1 expression was reduced in cells that survived for several days following treatment. This reduction was consistent with progression through the S-phase by surviving cells. Phosphorylation of RB1 was also reduced during the 10 days posttreatment (Fig. 4B), which suggests that cell cycle progression at G_1_–S is not facilitated by the effects of the canonical RB1-phosphorylation cascade (releasing E2F transcription factors; refs. 35, 48). The loss of the negative control that RB1 normally exerts on the cell cycle could contribute to skipping of G_1_–S and S checkpoints in surviving cells. As surviving cells resume proliferation, the expression of total RB1 returns to baseline. These observations are in line with data on polyploid giant cancer cells (PGCC) demonstrating that genes regulating cell cycle checkpoints are altered (49). Although a full explanation as to why total RB1 is decreased in drug-resilient cells remains opaque, we note that the HIF2α transcription factor has been shown to promote both RB1 (via the pro-S-phase RB1-E2F cascade) and AP-1 (e.g., complex members JUN) expression (50–52). Further elucidation of this mechanism is an avenue for future studies.

We show that the transcription factor HIF2α was highly upregulated in transiently polyploid and drug-resilient cancer cells, and that its downstream target genes and associated pathways are activated. HIF2 signaling appears to be applicable to many cell lines as hypoxic signaling was an upregulated pathway in ovarian PGCCs (49). Chromatin accessibility of *EPAS1* was increased in surviving breast cancer cells (HCC1806) at 10 DPT. HIF2α is typically degraded in the presence of oxygen. Our data show that cells surviving cisplatin treatment stabilized HIF2α in a hypoxia-independent manner, supported by the absence of hypoxia-responsive HIF1α expression in the same cell states.

HIF2α interacts with many regulators of the cell cycle and its stabilization in surviving cells posttreatment suggests that it may be critical for maintaining the cancer endocycle. AP-1 transcriptionally regulates cyclinD1 that, in a complex with CDK4/6, phosphorylates RB1, which initiates the cascade to release E2F that drives progression through the G_1_–S checkpoint. HIF2α interacts with AP-1, and both cyclinD1 and the AP-1 complex member JUN are downstream transcriptional targets of HIF2α (50–52). HIF2α has also been shown to promote entry into the S-phase in a RB1-independent manner by stabilizing the MYC/MAX complex, a G_1_–S promoting mechanism that parallels RB1/E2F (53–56). Thus, HIF2α can enable progression through G_1_–S to S- phase independently of RB1. MCM7 binds to HIF2α and promotes polyubiquitination and degradation, resulting in decreased levels of HIF2α (57). HIF2α signaling regulates embryonic development where the cell cycle oscillates between M and S, without gap phases; and embryonic gene sets have been seen in large cells (58, 59). We found that *MCM7* expression was decreased in the transient polyploid drug-resilient cells, with the expression decreasing in 10 DPT cells, while surviving cells undergo whole-genome duplication. The loss of MCM7 concomitant with HIF2α stabilization in endocycling cells suggests that HIF2α stabilization may also be associated with the waning of genome duplication. The observation that HIF2α KO did not completely ablate cell survival highlights the need to explore whether combinations of inhibitors together, or AP-1 inhibition in combination with HIF2α KO, would abrogate the entry or exit into the survival phenotype.

An alternative possibility is that these cells are entering a senescent-like state or somehow rewire their physiology toward another cell fate. Senescence was originally considered to be an irreversible cell cycle state, yet various studies have shown that it might well be reversible (60). Reversing senescence might be induced via manipulating critical regulators of senescence such as p53 or by altering the senescence-associated transcriptional program. HCC1806 cells are p53 null, while HCT116 cells are p53 proficient. Therefore, it would be expected that HCC1806 cell restart the cell cycle faster. Because this is not the case, this response is independent of p53. AP-1 opens the chromatin landscape to enhancers and is critical for the expression of the senescence associated transcriptional program. It has been shown to be important in early large-scale genome regulation; and AP-1 member expression was altered in all cell lines tested (61, 62). In addition, cells express DEC1 at 5 DPT and 10 DPT (Fig. 4C) which is a canonical marker of senescence (63). So, while the cell cycle RB1 checkpoint may have been ablated, AP-1 is still functioning to stop entry into mitosis. If surviving cells follow a similar pathway to reenter the cell cycle beyond 10 days posttreatment, depletion of AP-1 members could override the senescence transcriptional program.

**FIGURE 6:**
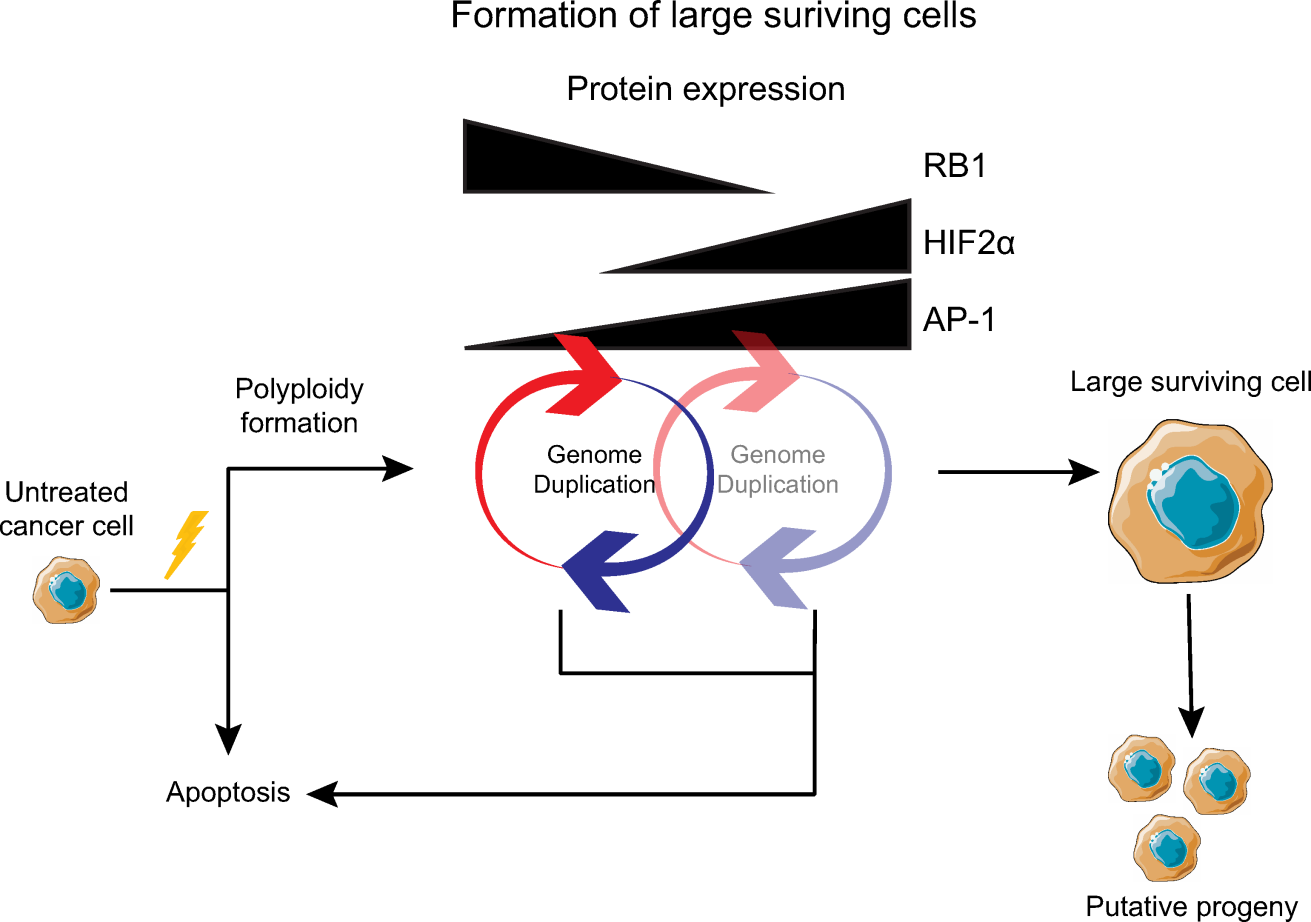
A model for surviving therapy. Cisplatin treatment induced whole-genome doubling without cell division resulting in large cells. Expression of HIF2α and AP-1 increased and appeared to help mediate cell survival. Eventually the cells ceased to increase in size and remained dormant for a period before undertaking cell division.

In summary, we suggest a conceptual model of therapy resistance that involves entry into a transient survival state characterized by an exit from the mitotic cycle and repeated whole-genome duplication in the absence of mitosis. Our data indicate that the upregulation of prosurvival pathways mediated by AP-1 and HIF2α supports a mechanism of whole-genome doubling via endocycling that could be therapeutically targeted (Fig. 6). This resistance model may represent an underappreciated mechanism of therapeutic resistance based on an evolutionary conserved stress response. Together, these results deepen our understanding of the formation of a survival phenotype and may contribute to developing novel approaches to overcome chemotherapy-induced resistance in cancer.

## Supplementary Material

Methods S1 Methods S1: Filtration of treated cells

Code S1 Code S1: Code for a Gaussian mixture model with two components.

Table S1 Table S1: LD50 values for cell lines after 72h cisplatin treatment.

Table S2 Table S2: Sequences of CRISPR gRNA to produce EPAS1 KO.

Table S3 Table S3: Antibodies used in the study.

Table S4 Table S4: Well counts for single cell colonies two months after cisplatin treatment.

Table S5 Table S5: scWGS heterogeneity scores.

Table S6 Table S6: Fish centromere counts.

Figure S1 Figure S1: Dose response curves for cell lines after 72h cisplatin treatment

Figure S2 Figure S2: Brightfield images of cell lines after treatment with LD50 cisplatin.

Figure S3 Figure S3: Cell diameter distributions for cell lines HCC1806, HCT116, and 786-0

Figure S4 Figure S4: Distribution fitting and cell population identification for cell line HCC1806.

Figure S5 Figure S5: Distribution fitting and Cell population identification for cell line HCT116

Figure S6 Figure S6: Distribution fitting and Cell population identification for cell line 786-0

Figure S7 Figure S7: Drug-resilient 786-0 cells exhibit 1-2 whole genome duplications with high fidelity

Figure S8 Figure S8: Pathways downregulated in HCC1806 cells surviving 10 DPT, as quantified with RNAseq and Reactome analysis.

Figure S9 Figure S9: Transcription factors regulating downregulated genes in HCC1806 cells surviving 10 DPT as quantified using RNAseq and CHEA3 analysis.

Figure S10 Figure S10: EPAS1 FPKM reads at different timepoints post treatment in HCC1806.

Figure S11 Figure S11: Survival of polyploid cells is reduced by the addition of NOTCH inhibitor during cisplatin treatment.

Figure S12 Figure S12: Survival of polyploid cells is reduced by the addition of NOTCH inhibitor during cisplatin treatment.

Figure S13 Figure S13: Effects of T2445 treatment on cellular proliferation at 10nM.

## References

[1] Seyfried TN , Huysentruyt LC. On the origin of cancer metastasis. Crit Rev Oncog 2013;18:43–73. 23237552 10.1615/critrevoncog.v18.i1-2.40 PMC3597235

[2] Housman G , Byler S, Heerboth S, Lapinska K, Longacre M, Snyder N, . Drug resistance in cancer: an overview. Cancers 2014;6:1769–92. 25198391 10.3390/cancers6031769 PMC4190567

[3] Hanahan D , Weinberg RA. Hallmarks of cancer: the next generation. Cell 2011;144:646–74. 21376230 10.1016/j.cell.2011.02.013

[4] Gillies RJ , Verduzco D, Gatenby RA. Evolutionary dynamics of carcinogenesis and why targeted therapy does not work. Nat Rev Cancer 2012;12:487–93. 22695393 10.1038/nrc3298 PMC4122506

[5] Aguadé-Gorgorió G , Kauffman S, Solé R. Transition therapy: tackling the ecology of tumor phenotypic plasticity. Bull Math Biol 2021;84:24. 34958403 10.1007/s11538-021-00970-9 PMC8712307

[6] Gupta PB , Pastushenko I, Skibinski A, Blanpain C, Kuperwasser C. Phenotypic plasticity: driver of cancer initiation, progression, and therapy resistance. Cell Stem Cell 2019;24:65–78. 30554963 10.1016/j.stem.2018.11.011 PMC7297507

[7] Burrell RA , Swanton C. Tumour heterogeneity and the evolution of polyclonal drug resistance. Mol Oncol 2014;8:1095–111. 25087573 10.1016/j.molonc.2014.06.005 PMC5528620

[8] Salmina K , Huna A, Kalejs M, Pjanova D, Scherthan H, Cragg MS, . The cancer aneuploidy paradox: in the light of evolution. Genes 2019;10:83. 30691027 10.3390/genes10020083 PMC6409809

[9] Storchova Z , Pellman D. From polyploidy to aneuploidy, genome instability and cancer. Nat Rev Mol Cell Biol 2004;5:45–54. 14708009 10.1038/nrm1276

[10] Yang F , Teoh F, Tan ASM, Cao Y, Pavelka N, Berman J. Aneuploidy enables cross-adaptation to unrelated drugs. Mol Biol Evol 2019;36:1768–82. 31028698 10.1093/molbev/msz104 PMC6657732

[11] Pienta KJ , Hammarlund EU, Austin RH, Axelrod R, Brown JS, Amend SR. Cancer cells employ an evolutionarily conserved polyploidization program to resist therapy. Semin Cancer Biol 2022;81:145–59. 33276091 10.1016/j.semcancer.2020.11.016

[12] Scholes DR , Paige KN. Plasticity in ploidy: a generalized response to stress. Trends Plant Sci 2015;20:165–75. 25534217 10.1016/j.tplants.2014.11.007

[13] Selmecki AM , Maruvka YE, Richmond PA, Guillet M, Shoresh N, Sorenson AL, . Polyploidy can drive rapid adaptation in yeast. Nature 2015;519:349–52. 25731168 10.1038/nature14187 PMC4497379

[14] Van de Peer Y , Ashman T-L, Soltis PS, Soltis DE. Polyploidy: an evolutionary and ecological force in stressful times. Plant Cell 2020;33:11–26. 10.1093/plcell/koaa015 PMC8136868 33751096

[15] Amend SR , Torga G, Lin K-C, Kostecka LG, de Marzo A, Austin RH, . Polyploid giant cancer cells: unrecognized actuators of tumorigenesis, metastasis, and resistance. Prostate 2019;79:1489–97. 31376205 10.1002/pros.23877 PMC6706309

[16] Yu CK , Sinclair WK. Polyploidy induced by X-rays during the cell cycle of Chinese hamster cells *in v**itro*. Radiat Res 1972;52:509–19. 4674993

[17] Lopez-Sánchez LM , Jimenez C, Valverde A, Hernandez V, Peñarando J, Martinez A, . CoCl2, a mimic of hypoxia, induces formation of polyploid giant cells with stem characteristics in colon cancer. PLoS One 2014;9:e99143. 24932611 10.1371/journal.pone.0099143 PMC4059626

[18] Jiang Y-H , Zhu Y, Chen S, Wang H-L, Zhou Y, Tang F-Q, . Re-enforcing hypoxia-induced polyploid cardiomyocytes enter cytokinesis through activation of β-catenin. Sci Rep 2019;9:17865. 31780774 10.1038/s41598-019-54334-4 PMC6883062

[19] Nair JS , Ho AL, Schwartz GK. The induction of polyploidy or apoptosis by the Aurora A kinase inhibitor MK8745 is p53-dependent. Cell Cycle 2012;11:807–17. 22293494 10.4161/cc.11.4.19323 PMC3318110

[20] Chen S , Liu M, Huang H, Li B, Zhao H, Feng XQ, . Heat stress-induced multiple multipolar divisions of human cancer cells. Cells 2019;8:888. 31412680 10.3390/cells8080888 PMC6721694

[21] Song Y , Zhao Y, Deng Z, Zhao R, Huang Q. Stress-induced polyploid giant cancer cells: unique way of formation and non-negligible characteristics. Front Oncol 2021;11:724781. 34527590 10.3389/fonc.2021.724781 PMC8435787

[22] Illidge TM , Cragg MS, Fringes B, Olive P, Erenpreisa JA. Polyploid giant cells provide a survival mechanism for p53 mutant cells after DNA damage. Cell Biol Int 2000;24:621–33. 10964452 10.1006/cbir.2000.0557

[23] Salmina K , Jankevics E, Huna A, Perminov D, Radovica I, Klymenko T, . Up-regulation of the embryonic self-renewal network through reversible polyploidy in irradiated p53-mutant tumour cells. Exp Cell Res 2010;316:2099–112. 20457152 10.1016/j.yexcr.2010.04.030

[24] Lagadec C , Vlashi E, Della Donna L, Dekmezian C, Pajonk F. Radiation-induced reprogramming of breast cancer cells. Stem Cells 2012;30:833–44. 22489015 10.1002/stem.1058 PMC3413333

[25] Puig PE , Guilly MN, Bouchot A, Droin N, Cathelin D, Bouyer F, . Tumor cells can escape DNA-damaging cisplatin through DNA endoreduplication and reversible polyploidy. Cell Biol Int 2008;32:1031–43. 18550395 10.1016/j.cellbi.2008.04.021

[26] Xu R , Wang K, Rizzi JP, Huang H, Grina JA, Schlachter ST, . 3-[(1S,2S,3R)-2,3-Difluoro-1-hydroxy-7-methylsulfonylindan-4-yl]oxy-5-fluorobenzonitrile (PT2977), a Hypoxia-inducible factor 2α (HIF-2α) inhibitor for the treatment of clear cell renal cell carcinoma. J Med Chem 2019;62:6876–93. 31282155 10.1021/acs.jmedchem.9b00719

[27] Ippolito MR , Martis V, Martin S, Tijhuis AE, Hong C, Wardenaar R, . Gene copy-number changes and chromosomal instability induced by aneuploidy confer resistance to chemotherapy. Dev Cell 2021;56:2440–54. 34352223 10.1016/j.devcel.2021.07.006

[28] Danecek P , Bonfield JK, Liddle J, Marshall J, Ohan V, Pollard MO, . Twelve years of SAMtools and BCFtools. Gigascience 2021;10:giab008. 33590861 10.1093/gigascience/giab008 PMC7931819

[29] Loh PR , Danecek P, Palamara PF, Fuchsberger C, A Reshef Y, K Finucane H, . Reference-based phasing using the haplotype reference consortium panel. Nat Genet 2016;48:1443–8. 27694958 10.1038/ng.3679 PMC5096458

[30] Zaccaria S , Raphael BJ. Characterizing allele- and haplotype-specific copy numbers in single cells with CHISEL. Nat Biotechnol 2021;39:207–14. 32879467 10.1038/s41587-020-0661-6 PMC9876616

[31] Bakker B , Taudt A, Belderbos ME, Porubsky D, Spierings DCJ, de Jong TV, . Single-cell sequencing reveals karyotype heterogeneity in murine and human malignancies. Genome Biol 2016;17:115. 27246460 10.1186/s13059-016-0971-7 PMC4888588

[32] Keenan AB , Torre D, Lachmann A, Leong AK, Wojciechowicz ML, Utti V, . ChEA3: transcription factor enrichment analysis by orthogonal omics integration. Nucleic Acids Res 2019;47:W212–24. 31114921 10.1093/nar/gkz446 PMC6602523

[33] Kim JA . Peroxisome metabolism in cancer. Cells 2020;9:1692. 32674458 10.3390/cells9071692 PMC7408135

[34] Harbour JW , Dean DC. Chromatin remodeling and Rb activity. Curr Opin Cell Biol 2000;12:685–9. 11063932 10.1016/s0955-0674(00)00152-6

[35] Rizzolio F , Lucchetti C, Caligiuri I, Marchesi I, Caputo M, Klein-Szanto AJ, . Retinoblastoma tumor-suppressor protein phosphorylation and inactivation depend on direct interaction with Pin1. Cell Death Differ 2012;19:1152–61. 22322860 10.1038/cdd.2011.202 PMC3374078

[36] Li Z , Bao S, Wu Q, Wang H, Eyler C, Sathornsumetee S, . Hypoxia-inducible factors regulate tumorigenic capacity of glioma stem cells. Cancer Cell 2009;15:501–13. 19477429 10.1016/j.ccr.2009.03.018 PMC2693960

[37] Holmquist-Mengelbier L , Fredlund E, Löfstedt T, Noguera R, Navarro S, Nilsson H, . Recruitment of HIF-1α and HIF-2α to common target genes is differentially regulated in neuroblastoma: HIF-2α promotes an aggressive phenotype. Cancer Cell 2006;10:413–23. 17097563 10.1016/j.ccr.2006.08.026

[38] Niklasson CU , Fredlund E, Monni E, Lindvall JM, Kokaia Z, Hammarlund EU, . Hypoxia inducible factor-2α importance for migration, proliferation, and self-renewal of trunk neural crest cells. Dev Dyn 2021;250:191–236. 32940375 10.1002/dvdy.253 PMC7891386

[39] Ko CY , Tsai MY, Tseng WF, Cheng CH, Huang CR, Wu JS, . Integration of CNS survival and differentiation by HIF2α. Cell Death Differ 2011;18:1757–70. 21546908 10.1038/cdd.2011.44 PMC3190110

[40] Kim C-J , Gonye ALK, Truskowski K, Lee C-F, Cho Y-K, Austin RH, . Nuclear morphology predicts cell survival to cisplatin chemotherapy. Neoplasia 2023;42:100906. 37172462 10.1016/j.neo.2023.100906 PMC10314150

[41] Hai T , Wolfgang CD, Marsee DK, Allen AE, Sivaprasad U. ATF3 and stress responses. Gene Expr 1999;7:321–35. 10440233 PMC6174666

[42] Li X , Zang S, Cheng H, Li J, Huang A. Overexpression of activating transcription factor 3 exerts suppressive effects in HepG2 cells. Mol Med Rep 2019;19:869–76. 30535500 10.3892/mmr.2018.9707 PMC6323204

[43] Tanaka Y , Nakamura A, Morioka MS, Inoue S, Tamamori-Adachi M, Yamada K, . Systems analysis of ATF3 in stress response and cancer reveals opposing effects on pro-apoptotic genes in p53 pathway. PLoS One 2011;6:e26848. 22046379 10.1371/journal.pone.0026848 PMC3202577

[44] Hackl C , Lang SA, Moser C, Mori A, Fichtner-Feigl S, Hellerbrand C, . Activating transcription factor-3 (ATF3) functions as a tumor suppressor in colon cancer and is up-regulated upon heat-shock protein 90 (Hsp90) inhibition. BMC Cancer 2010;10:668. 21129190 10.1186/1471-2407-10-668 PMC3003660

[45] Mahner S , Baasch C, Schwarz J, Hein S, Wölber L, Jänicke F, . C-Fos expression is a molecular predictor of progression and survival in epithelial ovarian carcinoma. Br J Cancer 2008;99:1269–75. 18854825 10.1038/sj.bjc.6604650 PMC2570515

[46] Casalino L , Talotta F, Cimmino A, Verde P. The Fra-1/AP-1 oncoprotein: from the “Undruggable” transcription factor to therapeutic targeting. Cancers 2022;14:1480. 35326630 10.3390/cancers14061480 PMC8946526

[47] Vleugel MM , Greijer AE, Bos R, van der Wall E, van Diest PJ. c-Jun activation is associated with proliferation and angiogenesis in invasive breast cancer. Hum Pathol 2006;37:668–74. 16733206 10.1016/j.humpath.2006.01.022

[48] Koirala N , Dey N, Aske J, De P. Targeting cell cycle progression in HER2+ breast cancer: an emerging treatment opportunity. Int J Mol Sci 2022;23:6547. 35742993 10.3390/ijms23126547 PMC9224522

[49] Adibi R , Moein S, Gheisari Y. Cisplatin-resistant ovarian cancer cells reveal a polyploid phenotype with remarkable activation of nuclear processes. Adv Biomed Res 2023;12:77. 37200756 10.4103/abr.abr_348_21 PMC10186044

[50] Liu Y , Lu C, Shen Q, Munoz-Medellin D, Kim H, Brown PH. AP-1 blockade in breast cancer cells causes cell cycle arrest by suppressing G1 cyclin expression and reducing cyclin-dependent kinase activity. Oncogene 2004;23:8238–46. 15378019 10.1038/sj.onc.1207889

[51] Labrecque MP , Takhar MK, Nason R, Santacruz S, Tam KJ, Massah S, . The retinoblastoma protein regulates hypoxia-inducible genetic programs, tumor cell invasiveness and neuroendocrine differentiation in prostate cancer cells. Oncotarget 2016;7:24284–302. 27015368 10.18632/oncotarget.8301 PMC5029701

[52] Bae WJ , Shin MR, Kang SK, Zhang J, Kim JY, Lee SC, . HIF-2 inhibition supresses inflammatory responses and osteoclastic differentiation in human periodontal ligament cells. J Cell Biochem 2015;116:1241–55. 25565665 10.1002/jcb.25078

[53] Gordan JD , Bertout JA, Hu CJ, Diehl JA, Simon MC. HIF-2alpha promotes hypoxic cell proliferation by enhancing c-myc transcriptional activity. Cancer Cell 2007;11:335–47. 17418410 10.1016/j.ccr.2007.02.006 PMC3145406

[54] Hoefflin R , Harlander S, Schafer S, Metzger P, Kuo F, Schonenberger D, . HIF-1α and HIF-2α differently regulate tumour development and inflammation of clear cell renal cell carcinoma in mice. Nat Commun 2020;11:4111. 32807776 10.1038/s41467-020-17873-3 PMC7431415

[55] Zhang H , Gao P, Fukuda R, Kumar G, Krishnamachary B, Zeller KI, . HIF-1 inhibits mitochondrial biogenesis and cellular respiration in VHL-deficient renal cell carcinoma by repression of C-MYC activity. Cancer Cell 2007;11:407–20. 17482131 10.1016/j.ccr.2007.04.001

[56] Santoni-Rugiu E , Falck J, Mailand N, Bartek J, Lukas J. Involvement of Myc activity in a G(1)/S-promoting mechanism parallel to the pRb/E2F pathway. Mol Cell Biol 2000;20:3497–509. 10779339 10.1128/mcb.20.10.3497-3509.2000 PMC85642

[57] Hubbi ME , Luo W, Baek JH, Semenza GL. MCM proteins are negative regulators of hypoxia-inducible factor 1. Mol Cell 2011;42:700–12. 21658608 10.1016/j.molcel.2011.03.029 PMC3131976

[58] Brantley SE , Di Talia S. Cell cycle control during early embryogenesis. Development 2021;148:dev193128. 34164654 10.1242/dev.193128 PMC8255047

[59] Salmina K , Vainshelbaum NM, Kreishmane M, Inashkina I, Cragg MS, Pjanova D, . The role of mitotic slippage in creating a “Female Pregnancy-like System” in a single polyploid giant cancer cell. Int J Mol Sci 2023;24:3237. 36834647 10.3390/ijms24043237 PMC9960874

[60] Shaban HA , Gasser SM. Dynamic 3D genome reorganization during senescence: defining cell states through chromatin. Cell Death Differ 2023 [Online ahead of print]. 10.1038/s41418-023-01197-y PMC11748698 37596440

[61] Krigerts J , Salmina K, Freivalds T, Zayakin P, Rumnieks F, Inashkina I, . Differentiating cancer cells reveal early large-scale genome regulation by pericentric domains. Biophys J 2021;120:711–24. 33453273 10.1016/j.bpj.2021.01.002 PMC7896032

[62] Martínez-Zamudio RI , Roux P-F, de Freitas JANLF, Robinson L, Doré G, Sun B, . AP-1 imprints a reversible transcriptional programme of senescent cells. Nat Cell Biol 2020;22:842–55. 32514071 10.1038/s41556-020-0529-5 PMC7899185

[63] Collado M , Gil J, Efeyan A, Guerra C, Schuhmacher AJ, Barradas M, . Tumour biology: senescence in premalignant tumours. Nature 2005;436:642. 16079833 10.1038/436642a

