# Supplementary material for: Drug-resilient Cancer Cell Phenotype Is Acquired via Polyploidization Associated with Early Stress Response Coupled to HIF2α Transcriptional Regulation
Source: Cancer Res Commun. 2024 Mar 7;4(3):691–705. doi: 10.1158/2767-9764.CRC-23-0396 (PMC10919208; doi:10.1158/2767-9764.CRC-23-0396)
Supplement: Methods S1 — Filtration of treated cells [file crc-23-0396-s01.docx]

Methods S1

Filtration and isolation of treated cells

After 72h treatment with chemotherapeutic or radiation, cell culture media was aspirated, and cells washed twice sterile PBS. Cells were lifted off the plate with 3 ml trypsin and placed into a 50 ml conical tube and neutralised with 17 ml of media (increase final volume if using multiple or large flasks). On a fresh 50 ml conical tube was added a connector ring and then the filter on top. 5 ml of fresh media was added onto the top of the mesh filter. A 10 ml syringe was inserted into the connector ring and gentle pulled to aspirate media through the mesh to prime the mesh filter. After the filter was primed the syringe was be removed. 5 ml of cells was added slowly to the filter and cells were allowed to pass through. 3 filters for each dish were used to ensure no clogging of the filter occured. Larger cells were be trapped the filter and cannot pass through. To isolate larger cells the mesh filter was upside down and placed onto a fresh 50 ml conical tube. The filter was washed with 10 ml of fresh media to force the large cells off the mesh and into the tube the large cells were seeded in a fresh dish or plate for downstream applications.

Gaussian mixture model for cell populations

Data was truncated at small diameters to remove cellular debris via the 'mle' function of MATLAB with the option 'TruncationBounds'. The minimal diameter of the experimental distributions was used as truncation value. Normality of generated data was subjected to a Kolmogorov-Smirnoff test (kstest). For data that did not fit a normal distribution a custom distribution was fitted to a manually defined truncated Gaussian mixture using either a with either two terms for the two components model, or three terms for three components model using the ‘mle’ function. The 'mle' function was used with the option 'pdf' to fit to a custom distribution, a manually defined truncated Gaussian mixture. This custom distribution was defined as a convex combination of normal distribution, with either two terms for the two components model, or three terms for three components model. The Kolmogorov-Smirnoff test was then used to test whether the sample could be generated by the fitted theoretical distributions. Code for fitting distributions in **Code S1**.

scWGS methods

1. The sequencing files (FASTQ) were aligned with a human reference genome (hg19/GRCh37 build) to generate alignment files (BAM) using the software BWA. BAM files that had less than 90% successfully aligned reads were removed from further analysis. The remaining number of BAM files are detailed in table S7.2.

2. Using GATK, a germline variant calling (HaplotypeCaller) was performed only on the control cells. After the variant calling, only heterozygous single nucleotide polimorphisms (SNPs) were kept, and quality filtered. Afterwards, Eagle was used for a haplotype phasing on the filtered SNPs.

3. All BAM files from each cell line are then merged into one BAM file and each read was barcoded by the software Chisel. The calculation of read-depth ratio is then performed.

4. Lastly, using Bcftools, problematic regions (i.e., centromeres, telomeres, and human genome blacklist regions) obtained from the ENCODE database (<https://www.encodeproject.org/annotations/ENCSR636HFF/>) are removed from the calls created by Chisel. And the plots are re-generated sorting the cells by replicates. All scripts containing the exact commands used for this analysis are publicly available on GitHub (<https://github.com/aboffelli/pacc-copy-number>).

Aneufinder methods

AneuFinder analysis Sequencing was performed using the reads generated by a NextSeq 500 machine (Illumina; up to 74, 77 or 78 cycles; single end).

1. The generated data were subsequently demultiplexed using sample-specific barcodes and changed into fastq files using bcl2fastq (Illumina; version 1.8.4). Reads were afterwards aligned to the human reference genome (GRCh38/hg38) using Bowtie2 (version 2.2.4; Langmead and Salzberg, 2012). Duplicate reads were marked with BamUtil (version 1.0.3; Jun et al., 2015). The aligned read data (bam files) were analyzed with a copy number calling algorithm called AneuFinder (Bakker, Taudt et al., 2016). Following GC correction and blacklisting of artefact-prone regions (extreme low or high coverage in control samples), libraries were analyzed using the dnacopy and edivisive copy number calling algorithms with variable width bins (average bin size = 1 Mb; step size = 500 kb) and breakpoint refinement (refine.breakpoints = TRUE).

2. Half of the samples were analyzed with the standard version of AneuFinder (from Bioconductor; version 1.14; HCC_CTR and S786_O_CTR) and the other half of the samples were analyzed with the developer version of AneuFinder (from GitHub; https://github.com/ataudt/aneufinder; version 1.7.4; constrained analysis; HCC_PACCs and S786_O_PACCs)^8^.

3. The ground ploidy for the samples that were analyzed with the developer version was constrained between certain values (min.ground.ploidy and max.ground.ploidy; HCC_PACCs: between 6.0 and 7.0; S786_O_PACCs: between 6.7 and 7.7). The samples were analyzed with an euploid reference (van den Bos et al., 2016). Results were afterwards curated by requiring a minimum concordance of 90 % (standard version) or 80 % (developer version) between the results of the two algorithms. Libraries with on average less than 10 reads per bin and per chromosome copy (~ 55,000 reads for a diploid genome) were discarded.

4. The aneuploidy score of each bin was calculated as the absolute difference between the observed copy number and the expected copy number when euploid. The score for each library was calculated as the weighted average of all the bins (size of the bin as weight) and the sample scores were calculated as the average of the scores of all libraries.

5. The heterogeneity score of each bin was calculated as the proportion of pairwise comparisons (cell 1 vs. cell 2, cell 1 vs cell 3, etc.) that showed a difference in copy number (e.g., cell 1: 2-somy and cell 2: 3-somy). The heterogeneity score of each sample was calculated as the weighted average of all the bin scores (size of the bin as weight).

6. The structural score of each library was calculated as the number of detected breakpoints (number of copy number transitions) divided by the total genome length in Mb (average number of breakpoints per Mb). The structural score of each sample was calculated as the average structural score of all libraries^8-11^.

RNAseq Methods

RNA degradation and contamination was monitored on 1% agarose gels. RNA purity was checked using the NanoPhotometer® spectrophotometer (IMPLEN, CA, USA). RNA integrity and quantitation were assessed using the RNA Nano 6000 Assay Kit of the Bioanalyzer 2100 system (Agilent Technologies, CA, USA).

A total amount of 1 μg RNA per sample was used as input material for the RNA sample preparations. Sequencing libraries were generated using NEBNext® UltraTM RNA Library Prep Kit for Illumina® (NEB, E7770, Ipswich, USA) following the manufacturer’s recommendations and index codes were added to attribute sequences to each sample. Briefly, mRNA was purified from total RNA using poly-T oligo-attached magnetic beads. Fragmentation was carried out using divalent cations under elevated temperature in NEBNext First Strand Synthesis Reaction Buffer (5X) or by using sonication with Diagenode bioruptor Pico for breaking RNA strands. First strand cDNA was synthesized using random hexamer primer and M-MuLV Reverse Transcriptase (RNase H-). Second strand cDNA synthesis was subsequently performed using DNA Polymerase I and RNase H. Remaining overhangs were converted into blunt ends via exonuclease/polymerase activities. After adenylation of 3’-ends of DNA fragments, NEBNext Adaptors with hairpin loop structure were ligated to prepare for hybridization. To select cDNA fragments of preferentially 150~200 bp in length, the library fragments were purified with AMPure XP system (Beckman Coulter, Beverly, USA). Then 3 μl USER Enzyme (NEB, M5505, Ipswich, USA) was used with size-selected, adaptor-ligated cDNA at 37 °C for 15 minutes followed by 5 minutes at 95 °C prior to PCR. PCR was performed with Phusion High-Fidelity DNA polymerase, Universal PCR primers and Index (NEB, E7335, USA) Primer. Finally, PCR products were purified (AMPure XP system) and library quality was assessed on the Agilent Bioanalyzer 2100 system.

Raw data (raw reads) of FASTQ format were firstly processed through fastp^1^. In this step, clean data (clean reads) were obtained by removing reads containing adapter and poly-N sequences and reads with low quality from raw data. At the same time, Q20, Q30, and GC content of the clean data were calculated. All the downstream analyses were based on the clean data with high quality.

Reference genome and gene model annotation files were downloaded from the genome website browser (NCBI/UCSC/Ensembl) directly. Paired-end clean reads were aligned to the reference genome using the Spliced Transcripts Alignment to a Reference (STAR) software^2^, which is based on a previously undescribed RNA-seq alignment algorithm that uses sequential maximum mappable seed search in uncompressed suffix arrays followed by seed clustering and stitching procedure. STAR exhibits better alignment precision and sensitivity than other RNA-seq aligners for both experimental and simulated data^2^.

FeatureCounts was used to count the read numbers mapped of each gene. And then RPKM of each gene was calculated based on gene length and reads count mapped to this gene. Reads Per Kilobase of exon model per Million (RPKM) mapped reads, considers the effect of sequencing depth and gene length for the reads count at the same time, and is currently the most commonly used method for estimating gene expression levels^3^.

For DESeq2 with biological replicates: Differential expression analysis between two conditions/groups (three biological replicates per condition) was performed using the DESeq2 R package^4^. DESeq2 provides statistical routines for determining differential expression in digital gene expression data using a model based on the negative binomial distribution. The resulting *p* values were adjusted using the Benjamini and Hochberg’s approach for controlling the False Discovery Rate (FDR). Genes with an adjusted *p* value < 0.05 found by DESeq2 were assigned as differentially expressed.

For edgeR without biological replicates: Prior to differential gene expression analysis, for each sequenced library, the read counts were adjusted by Trimmed Mean of Mvalues (TMM) through one scaling normalized factor^5^. Differential expression analysis of two conditions was performed using the edgeR R package. The *p* values were adjusted using the Benjamini and Hochberg methods. Corrected *p* value of 0.005 and |log2 (Fold Change)| of 1 were set as the threshold for significantly differential expression.

Alternative splicing analysis was performed by the software rMATS^6^, a statistical method for robust and flexible detection of differential AS from replicate RNA-Seq data. It identifies alternative splicing events corresponding to all major types of alternative splicing patterns and calculates the *p* value and FDR for differential splicing. These types include exon skipping (SE), alternative 5′ splice sites (A5SS), alternative 3′ splice sites (A3SS), mutually exclusive exons (MXE), and retained introns (RI).

Firstly, Picard tools and Samtools were used to sort, mark duplicated reads, and reorder the bam alignment results of each sample. Then the tool HaplotypeCaller in GATK software was used to perform variant discovery. Raw VCF files were filtered with GATK standard filter method and other parameters (cluster: 3; WindowSize: 35; QD < 2.0 or FS >30.0). Finally, ANNOVAR was used to functionally annotate genetic variants detected from diverse genomes against UCSC Genome Browser, dbSNP database, the 1000 Genomes Project, and so on^7^.

For human and mouse data, we use STAR-Fusion to identify fusion genes in tumour samples. By enabling the STAR option to report the chimeric read alignments, these data will be readily available for running STAR-Fusion, and quickly provide access to lists of candidate fusion transcripts. Those fusions that pass the filters are reported in a tab-delimited summary file identifying the fusion pairs, the inferred fusion breakpoint (chromosomal exon boundaries), counts of supporting split reads and spanning fragments, and identification of the RNA-Seq reads that support the fusion prediction. The parameters of "--min_junction_reads" and "--min_sum_frags" were assigned default values.

ATACseq Methods

1. Sequence Analysis: The paired-end 42 bp sequencing reads (PE42) generated by Illumina sequencing (using NextSeq 500) were mapped to the genome using the BWA algorithm with default settings (“bwa mem”). Alignment information for each read was stored in the BAM format. Only reads that passed Illumina’s purity filter, aligned with no more than 2 mismatches, and mapped uniquely to the genome were used in the subsequent analysis. In addition, duplicate reads (“PCR duplicates”) were removed.

2. Peak Finding: Genomic regions with high levels of transposition/tagging events were determined using the MACS2 peak calling algorithm (Zhang et al., Genome Biology 2008, 9:R137). Since both reads (tags) from paired-end sequencing represent transposition events, both reads were used for peak-calling but treated as single, independent reads. Note that we used the generic term “Interval” to describe the genomic regions identified by MACS2. Intervals were defined by the chromosome number and a start and end coordinate.

3. Determination of Fragment Density: To identify the density of transposition events along the genome, the genome was divided into 32 bp bins and the number of fragments in each bin was determined. For this purpose, reads were extended to 200 bp, which is close to the average length of the sequenced library inserts. *In silico* extension also helped to smooth the data. This information (“signal map”; histogram of fragment densities) was stored in a bigWig file, which can be visualized in genome browsers (see Section VI.). BigWig files are also the basis of the peak metrics in the Active Motif analysis program described below.

4. Normalization: In the default analysis, the tag number of all samples was reduced (by random sampling) to the number of tags present in the smallest sample. When samples were not directly comparable or in cases of projects with outlier samples, this strategy was either forgone or could potentially be customized to fit the experimental design.

5. Merged Region Analysis: To compare peak metrics between 2 or more samples, overlapping Intervals (orange bars in diagram on next page) were grouped into “Merged Regions” (green bars), which were defined by the start coordinate of the most upstream Interval and the end coordinate of the most downstream Interval (= union of overlapping Intervals; “merged peaks”). In locations where only one sample had an Interval, this Interval defined the Merged Region. The use of Merged Regions was necessary because the locations and lengths of Intervals were rarely exactly the same when comparing different samples. Furthermore, with this approach fragment density values could be obtained even for samples for which no peak was called.
