# Supplementary material for: Drug-resilient Cancer Cell Phenotype Is Acquired via Polyploidization Associated with Early Stress Response Coupled to HIF2α Transcriptional Regulation
Source: Cancer Res Commun. 2024 Mar 7;4(3):691–705. doi: 10.1158/2767-9764.CRC-23-0396 (PMC10919208; doi:10.1158/2767-9764.CRC-23-0396)
Supplement: Code S1 — Code for a Gaussian mixture model with two components. [file crc-23-0396-s02.docx]

**Code S1.** Code for a Gaussian mixture model with two components.

clear all

close all

%%%%%%%%%%%%%%%%Loading Data%%%%%%%%%%%%%%%%%%

dataType='CTL' %Choosing the sample

data=readtable('Flowcam diameters.xlsx'); %Loading data

%%%%%%%%%%%%%%%%Building Result Folder%%%%%%%%%%%%%%%%%%

pathResult=strcat('Results/',dataType);

dataVect=evalin('base',['data.' dataType])

dataVect=dataVect(~isnan(dataVect)); %Cleaning data

truncVal=min(dataVect);

pathResult=strcat(pathResult,'/')

if ~exist(pathResult,'dir')

mkdir(pathResult);

end

%%%%%%%%%%%%%%%%Testing normal distribution null hypothesis%%%%%%%%%%%%%%%%%%

testName='kstest' %Choosing a statistical test

[h,p]=statTest(dataVect,testName) %Performing the test

legendFontSize=14 %Legend font size

%%%%%%%%%%%%%%%%Figure1%%%%%%%%%%%%%%%%%%

figure(1)

hist=histogram(dataVect,'Normalization','pdf');%Plotting data in a histogram

saveas(gcf,strcat(pathResult,'Histo.png'));

%%%%%%%%%%%%%%%%Fit to normal distribution%%%%%%%%%%%%%%%%%%

xgrid = linspace(min(dataVect),max(dataVect),200);

pd = fitdist(dataVect,'Normal'); %Fit to a normal distribution

%%%%%%%%%%%%%%%%Figure2%%%%%%%%%%%%%%%%%%

figure(2)

hist=histogram(dataVect,'Normalization','pdf');

hold on

plot(xgrid,pdf(pd,transpose(xgrid)),'LineWidth',2);%Plotting fitted normal distribution against data in a histogram

if (p<0.05)

lgd=legend('data',strcat('normal distribution fit, ',testName,' rejected p=',string(p)))

lgd.FontSize = legendFontSize

legend boxoff

else

lgd=legend('data',strcat('normal distribution fit, ',testName,' p=',string(p)))

lgd.FontSize = legendFontSize

legend boxoff

end

saveas(gcf,strcat(pathResult,'FitNormal.png'));

%%%%%%%%%%%%%%%%Fit to truncated normal distribution%%%%%%%%%%%%%%%%%%

[lambdaHat,lambdaCI] = mle(dataVect,'Distribution','Normal', ...

'TruncationBounds',[truncVal Inf]) %Parameters estimation

pd = makedist('Normal',lambdaHat(1),lambdaHat(2)) %Defining the distribution object

t = truncate(pd,truncVal,inf) %Truncate distribution

test_cdf=[dataVect cdf(t,dataVect)] %Defining the cumulative distribution function

[h2,p2] = kstest(dataVect,'CDF',test_cdf) %Statistical test checking hypothesis that experimental distribution comes from the fitted truncated gaussian mixture distribution

figure(3)

hist=histogram(dataVect,'Normalization','pdf');

hold on

plot(xgrid,pdf(t,transpose(xgrid)),'LineWidth',2);%Plotting fitted truncated normal distribution against data in a histogram

if (p2<0.05)

lgd=legend('data',strcat('truncated normal distribution fit, ',testName,' rejected p=',string(p2)))

lgd.FontSize = legendFontSize

legend boxoff

else

lgd=legend('data',strcat('truncated normal distribution fit, ',testName,' p=',string(p2)))

lgd.FontSize = legendFontSize

legend boxoff

end

saveas(gcf,strcat(pathResult,'FitTruncNormal.png'));

%%%%%%%%%%%%%%%%Initial parameter values of the two components Gaussian mixture%%%%%%%%%%%%%%%%%%

pStart = .5; %Initial first component proportion

%muStart = [.75*lambdaHat(1) 1.25*lambdaHat(1)] %Initial Average values

%muStart = [20 25] %Initial Average values

muStart = [25 35] %Initial Average values

%muStart = [33 40] %Initial Average values

%muStart = [25 30] %Initial Average values

%muStart = [35 55] %Initial Average values

%muStart = [56 58] %Initial Average values

%sigmaStart = lambdaHat(2) %Initial Std values

sigmaStart = lambdaHat(2) %Initial Std values

start = [pStart muStart sigmaStart sigmaStart]; %Initial vector values

%%%%%%%%%%%%%%%%Initial parameter values of the three components Gaussian mixture%%%%%%%%%%%%%%%%%%

p1Start3 = .25; %Initial first component proportion

p2Start3 = .5; %Initial second component proportion

%muStart3 = [20 25 30] %Initial Average values

%muStart3 = [25 30 40] %Initial Average values

muStart3 = [35 45 55] %Initial Average values

%muStart3 = [20 35 50] %Initial Average values

sigmaStart3 = lambdaHat(2) %Initial Std values

start3 = [p1Start3 p2Start3 muStart3 sigmaStart3 sigmaStart3 sigmaStart3]; %Initial vector values

%%%%%%%%%%%%%%%%Defining the truncated Gaussian mixture functions%%%%%%%%%%%%%%%%%%

pdf_normmixture = @(x3,p,mu1,mu2,sigma1,sigma2) ...

(p*normpdf(x3,mu1,sigma1) + (1-p)*normpdf(x3,mu2,sigma2));

pdf_normmixtureTrunc = @(x3,p,mu1,mu2,sigma1,sigma2) ...

makenormmixtureTrunc(x3,p,mu1,mu2,sigma1,sigma2,truncVal); %Two components function

pdf_normmixtureTrunc3 = @(x1,p1,p2,mu1,mu2,mu3,sigma1,sigma2,sigma3) ...

makenormmixtureTrunc3(x1,p1,p2,mu1,mu2,mu3,sigma1,sigma2,sigma3,truncVal); %Three components function

%%%%%%%%%%%%%%%%Defining the parameter boundaries of the fitting algorithm%%%%%%%%%%%%%%%%%%

lb = [0 truncVal truncVal 0 0]; %LowerBound of the two components truncated Gaussian mixture

ub = [1 Inf Inf Inf Inf]; %%UpperBound of the two components truncated Gaussian mixture

lb3 = [0 0 truncVal truncVal truncVal 0 0 0]; %LowerBound of the three components truncated Gaussian mixture

ub3 = [1 1 Inf Inf Inf Inf Inf Inf]; %UpperBound of the three components truncated Gaussian mixture

%%%%%%%%%%%%%%%%Defining the parameter boundaries of the fitting algorithm%%%%%%%%%%%%%%%%%%

options = statset('MaxIter',3000,'MaxFunEvals',6000); %Fitting settings

paramEsts = mle(dataVect,'pdf',pdf_normmixtureTrunc,'Start',start, ...

'LowerBound',lb,'UpperBound',ub,'Options',options); %Parameter estimation of the two components truncated Gaussian mixture

paramEsts3 = mle(dataVect,'pdf',pdf_normmixtureTrunc3,'Start',start3, ...

'LowerBound',lb3,'UpperBound',ub3,'Options',options); %Parameter estimation of the three components truncated Gaussian mixture

Mu(1,1)=paramEsts(2);

Mu(2,1)=paramEsts(3);

Sigma(1,1,1)=paramEsts(4);

Sigma(1,1,2)=paramEsts(5);

Proportion(1,1)=paramEsts(1);

Proportion(1,2)=1-paramEsts(1);

%%%%%%%%%%%%%%%%Defining Gaussian mixture functions with calibrated parameter values%%%%%%%%%%%%%%%%%%

pdf_normmixtureTruncFitted=@(x3)pdf_normmixtureTrunc(x3,paramEsts(1),paramEsts(2),paramEsts(3),paramEsts(4),paramEsts(5));

cdf_normmixtureTrunc=@(x3)integral(pdf_normmixtureTruncFitted,truncVal,x3);

pdf_normmixtureTruncFitted3=@(x3)pdf_normmixtureTrunc3(x3,paramEsts3(1),paramEsts3(2),paramEsts3(3),paramEsts3(4),paramEsts3(5),paramEsts3(6),paramEsts3(7),paramEsts3(8));

cdf_normmixtureTrunc3=@(x3)integral(pdf_normmixtureTruncFitted3,truncVal,x3);

%%%%%%%%%%%%%%%%Build value vectors from two component fitted Gaussian mixture%%%%%%%%%%%%%%%%%%

for i=1:length(dataVect)

cdf_Vect(i)=cdf_normmixtureTrunc(dataVect(i));

end

cdf_Vect=transpose(cdf_Vect);

%%%%%%%%%%%%%%%%Build value vectors from three component fitted Gaussian mixture%%%%%%%%%%%%%%%%%%

for i=1:length(dataVect)

cdf_Vect3(i)=cdf_normmixtureTrunc3(dataVect(i));

end

cdf_Vect3=transpose(cdf_Vect3);

%%%%%%%%%%%%%%%%Statistical test to assess whether it is likely that the fitted distrubtions could generate the experimental sample%%%%%%%%%%%%%%%%%%

test_cdf2=[dataVect cdf_Vect]; %Defining the cumulative distribution function

[h4,p4] = kstest(dataVect,'CDF',test_cdf2); %Statistical test checking hypothesis that experimental distribution comes from the fitted gaussian mixture distribution

test_cdf3=[dataVect cdf_Vect3]; %Defining the cumulative distribution function

[h5,p5] = kstest(dataVect,'CDF',test_cdf3); %Statistical test checking hypothesis that experimental distribution comes from the fitted three components gaussian mixture distribution

%%%%%%%%%%%%%%%%Figure4 Truncated Two components Mixture Fit%%%%%%%%%%%%%%%%%%

xgrid = linspace(min(dataVect),max(dataVect),200);

pdfgrid = pdf_normmixtureTrunc(xgrid, ...

paramEsts(1),paramEsts(2),paramEsts(3),paramEsts(4),paramEsts(5));

figure(4)

hist=histogram(dataVect,'Normalization','pdf');

hold on

plot(xgrid,pdfgrid,'LineWidth',2)

if (p4<0.05)

lgd=legend('data',strcat('Truncated Gaussian mixture fit, kstest rejected p=',string(p4)))

lgd.FontSize = legendFontSize

legend boxoff

else

lgd=legend('data',strcat('Truncated Gaussian mixture fit, kstest p=',string(p4)))

lgd.FontSize = legendFontSize

legend boxoff

end

saveas(gcf,strcat(pathResult,'FitMixtureTrunc.png'));

%%%%%%%%%%%%%%%%Figure5 Truncated Three components Mixture Fit%%%%%%%%%%%%%%%%%%

figure(5)

xgrid = linspace(min(dataVect),max(dataVect),200);

pdfgrid = pdf_normmixtureTrunc3(xgrid, ...

paramEsts3(1),paramEsts3(2),paramEsts3(3),paramEsts3(4),paramEsts3(5),paramEsts3(6),paramEsts3(7),paramEsts3(8));

hist=histogram(dataVect,'Normalization','pdf');

hold on

plot(xgrid,pdfgrid,'LineWidth',2)

if (p5<0.05)

lgd=legend('data',strcat('Truncated Gaussian mixture fit (3 componenents), kstest rejected p=',string(p5)),'Location','best')

lgd.FontSize = legendFontSize

legend boxoff

else

lgd=legend('data',strcat('Truncated Gaussian mixture fit (3 componenents), kstest p=',string(p5)),'Location','northwest')

lgd.FontSize = legendFontSize

legend boxoff

end

saveas(gcf,strcat(pathResult,'FitMixtureTrunc3.png'));

%%%%%%%%%%%%%%%%Defining Separate components of two components Gaussian mixture%%%%%%%%%%%%%%%%%%

n1_2=makedist('normal',paramEsts(2),paramEsts(4));%First Normal distribution component

n2_2=makedist('normal',paramEsts(3),paramEsts(5));%Second Normal distribution component

t1_2 = truncate(n1_2,truncVal,inf);%Truncate

t2_2 = truncate(n2_2,truncVal,inf);%Truncate

%%%%%%%%%%%%%%%%Figure6 Plotting the two components of the two component mixture separately%%%%%%%%%%%%%%%%%%

figure(6)

plot(xgrid,pdf(t1_2,transpose(xgrid)));

hold on

plot(xgrid,pdf(t2_2,transpose(xgrid))); %Plotting the two components of the Gaussian mixture

lgd=legend(strcat('component 1, diameter=',string(paramEsts(2)),', standard deviation=',string(sqrt(paramEsts(4))),', proportion=',string(paramEsts(1))),strcat('component 2, diameter=',string(paramEsts(3)),', standard deviation=',string(sqrt(paramEsts(5))),', proportion=',string(1-paramEsts(1))),'Location','northwest')

lgd.FontSize = legendFontSize

legend boxoff

saveas(gcf,strcat(pathResult,'FitMixtureTruncTwoDistrib.png'));

%%%%%%%%%%%%%%%%Defining Separate components of three components Gaussian mixture%%%%%%%%%%%%%%%%%%

n1_3=makedist('normal',paramEsts3(3),paramEsts3(6));%First Normal distribution component

n2_3=makedist('normal',paramEsts3(4),paramEsts3(7));%Second Normal distribution component

n3_3=makedist('normal',paramEsts3(5),paramEsts3(8));%Third Normal distribution component

t1_3 = truncate(n1_3,truncVal,inf);%Truncate

t2_3 = truncate(n2_3,truncVal,inf);%Truncate

t3_3 = truncate(n3_3,truncVal,inf);%Truncate

p1_3=paramEsts3(1);%Proportion 1

p2_3=paramEsts3(2)*(1-paramEsts3(1));%Proportion 2

p3_3=1-p1_3-p2_3;%Proportion 3

%%%%%%%%%%%%%%%%Figure7 Plotting the three components of the three component mixture separately%%%%%%%%%%%%%%%%%%

figure(7)

plot(xgrid,pdf(t1_3,transpose(xgrid)));

hold on

plot(xgrid,pdf(t2_3,transpose(xgrid))); %Plotting the two components of the Gaussian mixture

hold on

plot(xgrid,pdf(t3_3,transpose(xgrid))); %Plotting the two components of the Gaussian mixture

lgd=legend(strcat('component 1, diameter=',string(paramEsts3(3)),', standard deviation=',string(sqrt(paramEsts3(6))),', proportion=',string(p1_3)),strcat('component 2, diameter=',string(paramEsts3(4)),', standard deviation=',string(sqrt(paramEsts3(7))),', proportion=',string(p2_3)),strcat('component 3, diameter=',string(paramEsts3(5)),', standard deviation=',string(sqrt(paramEsts3(8))),', proportion=',string(p3_3)))

lgd.FontSize = legendFontSize

legend boxoff

saveas(gcf,strcat(pathResult,'FitMixtureTruncThreeDistrib.png'));

%%%%%%%%%%%%%%%%Statistical Test Function%%%%%%%%%%%%%%%%%%

function [h,p]=statTest(data,testName)

CenteredData=data-mean(data);

if (isequal(testName,'ttest'))

[h,p]=ttest(CenteredData)

elseif (isequal(testName,'ttestMean'))

[h,p]=ttest(data,mean(data))

elseif (isequal(testName,'kstest'))

[h,p]=kstest(CenteredData/std(data))

elseif (isequal(testName,'Lillietest'))

[h,p]=lillietest(data)

elseif (isequal(testName,'ztest'))

[h,p]=ztest(CenteredData,0,std(CenteredData))

elseif (isequal(testName,'adtest'))

[h,p]=adtest(data)

else

[h,p]=kstest(CenteredData/std(data))

end

[h,p]=kstest(CenteredData/std(data))

end

function Ind=Indicator(x,a)

Ind=0

if (x>a)

Ind=1

end

end

%%%%%%%%%%%%%%%%Function defining truncated two components Gaussian mixture distribution%%%%%%%%%%%%%%%%%%

function normmixture=makenormmixtureTrunc(x1,p,mu1,mu2,sigma1,sigma2,truncVal)

n1=makedist('normal',mu1,sigma1);

n2=makedist('normal',mu2,sigma2);

t1 = truncate(n1,truncVal,inf);

t2 = truncate(n2,truncVal,inf);

normmixture=(p*pdf(t1,x1)+(1-p)*pdf(t2,x1))./(p*(1-cdf(t1,truncVal))+(1-p)*(1-cdf(t2,truncVal)));

end

%%%%%%%%%%%%%%%%Function defining truncated three components Gaussian mixture distribution%%%%%%%%%%%%%%%%%%

function normmixture3=makenormmixtureTrunc3(x1,p1,p2,mu1,mu2,mu3,sigma1,sigma2,sigma3,truncVal)

n1=makedist('normal',mu1,sigma1);

n2=makedist('normal',mu2,sigma2);

n3=makedist('normal',mu3,sigma3);

t1 = truncate(n1,truncVal,inf);

t2 = truncate(n2,truncVal,inf);

t3 = truncate(n3,truncVal,inf);

normmixture3=(p1*pdf(t1,x1)+p2*(1-p1)*pdf(t2,x1)+(1-(p2*(1-p1)+p1))*pdf(t3,x1))./(p1*(1-cdf(t1,truncVal))+p2*(1-p1)*(1-cdf(t2,truncVal))+(1-(p2*(1-p1)+p1))*(1-cdf(t3,truncVal)));

end
