# Supplementary material for: Drug-resilient Cancer Cell Phenotype Is Acquired via Polyploidization Associated with Early Stress Response Coupled to HIF2α Transcriptional Regulation
Source: Cancer Res Commun. 2024 Mar 7;4(3):691–705. doi: 10.1158/2767-9764.CRC-23-0396 (PMC10919208; doi:10.1158/2767-9764.CRC-23-0396)
Supplement: Table S1 — LD50 values for cell lines after 72h cisplatin treatment. [file crc-23-0396-s03.docx]

**Table S1.** LD50 values for the cell lines after treatment with cisplatin for 72h.
