# Supplementary material for: Drug-resilient Cancer Cell Phenotype Is Acquired via Polyploidization Associated with Early Stress Response Coupled to HIF2α Transcriptional Regulation
Source: Cancer Res Commun. 2024 Mar 7;4(3):691–705. doi: 10.1158/2767-9764.CRC-23-0396 (PMC10919208; doi:10.1158/2767-9764.CRC-23-0396)
Supplement: Table S2 — Sequences of CRISPR gRNA to produce EPAS1 KO. [file crc-23-0396-s04.docx]

**Table S2**. gRNA sequence for *EPAS1* KO

| Target | Sequence |
| --- | --- |
| Control | CGCGAUUGCGCGAAUAUAUU |
| EPAS1 | ACCCTATATCCCCATGGAC |

Note: gRNA sequence for *EPAS1* KO
