# Supplementary material for: Drug-resilient Cancer Cell Phenotype Is Acquired via Polyploidization Associated with Early Stress Response Coupled to HIF2α Transcriptional Regulation
Source: Cancer Res Commun. 2024 Mar 7;4(3):691–705. doi: 10.1158/2767-9764.CRC-23-0396 (PMC10919208; doi:10.1158/2767-9764.CRC-23-0396)
Supplement: Table S3 — Antibodies used in the study. [file crc-23-0396-s05.docx]

**Table S3** Antibodies used in the study.

| Target | Manufacturer | Catalog number | RRID |
| --- | --- | --- | --- |
| ACTIN | Biorad | 12004163 | AB_2861334 |
| NUR77 | Cell Signalling | 3960S | AB_2153738 |
| HIC1 | Sigma-Aldrich | 16074115 | AB_1079054 |
| MCM7 | Cell Signalling | 3735 | AB_2142705 |
| RB1 | Cell Signalling | 9309 | AB_823629 |
| RB1 (807) | Cell Signalling | 8516 | AB_11178658 |
| RB1 (780) | Cell Signalling | 8180 | AB_10950972 |
| RB1 (795) | Cell Signalling | 9301 | AB_330013 |
| HIF2 | Abcam | ab20654 | AB_2293452 |
| SERPINB9 | Abcam | ab150400 | NA |
| VEGFA | Santa Cruz Biotech | sc-7269 | AB_628430 |
| DEC1 | Novus Biological | NB100-1800SS | AB_1852832 |
| VHL | Abcam | ab140989 | AB_2934140 |
| PHD1 | Santa Cruz Biotech | sc-46024 | AB_2096864 |
| PHD2 | Fisher | PA116525 | AB_568563 |
| PHD3 | Fisher | PA116526 | AB_2293343 |
| FOS | Fisher | 15308010 | AB_2538547 |
| FOSL1 | Cell Signalling | 5281S | AB_10557418 |
| cUN | Cell Signalling | 9165S | AB_2130165 |
| FOSL2 | Thermofisher | 2355-MSM1-P1 | NA |
| FOSB | Cell Signalling | 2263S | AB_2106900 |
| ATF3 | Cell Signalling | 33593 | AB_2799039 |
