# Supplementary material for: Drug-resilient Cancer Cell Phenotype Is Acquired via Polyploidization Associated with Early Stress Response Coupled to HIF2α Transcriptional Regulation
Source: Cancer Res Commun. 2024 Mar 7;4(3):691–705. doi: 10.1158/2767-9764.CRC-23-0396 (PMC10919208; doi:10.1158/2767-9764.CRC-23-0396)
Supplement: Table S5 — scWGS heterogeneity scores. [file crc-23-0396-s07.docx]

**Table S5** Heterogeneity scores for scWGS.

| Sample | Heterogeneity score |
| --- | --- |
| HCC1806 CTL | 0.441087 |
| HCC1806 5 DPT | 0.082794 |
| 786-0 CTL | 0.237646 |
| 786-0 5 DPT | 0.066559 |
