# Supplementary material for: Drug-resilient Cancer Cell Phenotype Is Acquired via Polyploidization Associated with Early Stress Response Coupled to HIF2α Transcriptional Regulation
Source: Cancer Res Commun. 2024 Mar 7;4(3):691–705. doi: 10.1158/2767-9764.CRC-23-0396 (PMC10919208; doi:10.1158/2767-9764.CRC-23-0396)

**Figure S1**. Dose response curves for cell lines after 72h cisplatin treatment. Cells were plated into 6 well dishes and exposed to cisplatin for 72h with a minimum of three technical replicates per concentration with 3 biological replicates performed. Cell viability was measured with the trypan blue and counted using BioRad TC20 cell counter according to the manufacturer’s instructions. Absolute viability values were converted to percentage viability versus control treatment, and then nonlinear fit of log(inhibitor) versus response (three parameters) was performed in GraphPad Prismv9.0 to obtain the LD50 values.


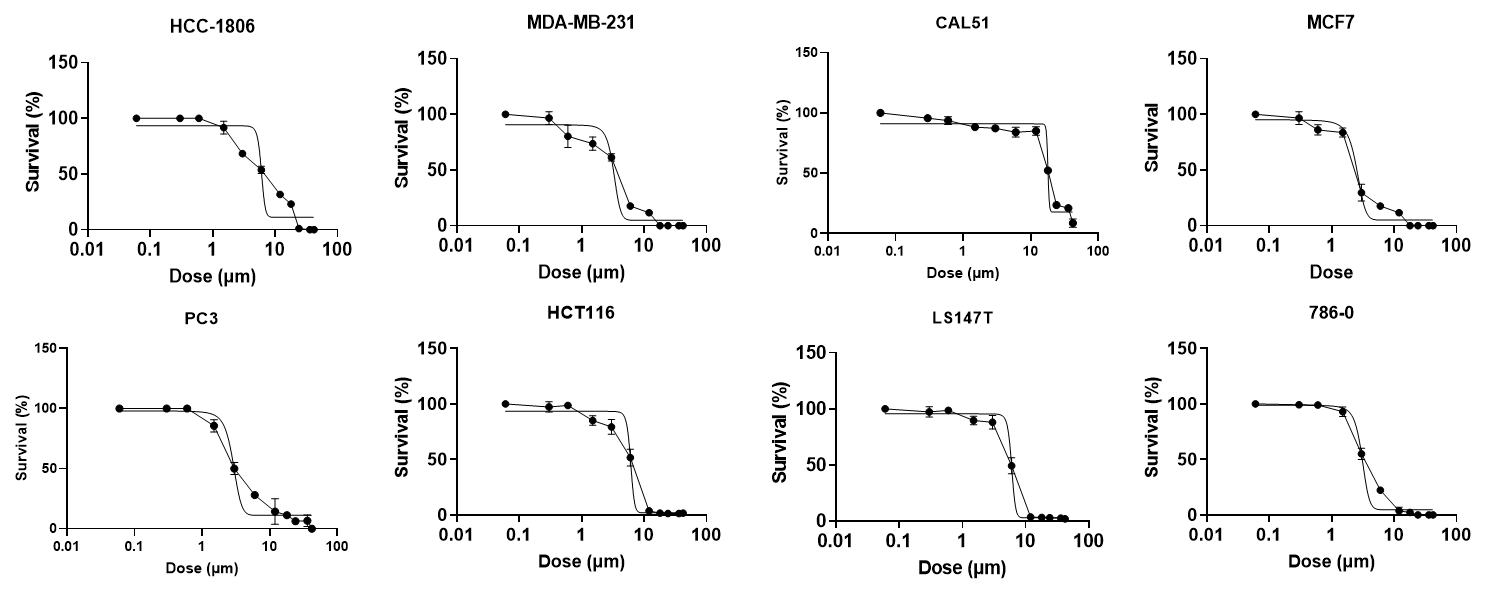

Supplement: Figure S1 — Dose response curves for cell lines after 72h cisplatin treatment [file crc-23-0396-s09.docx]
