# Supplementary material for: Drug-resilient Cancer Cell Phenotype Is Acquired via Polyploidization Associated with Early Stress Response Coupled to HIF2α Transcriptional Regulation
Source: Cancer Res Commun. 2024 Mar 7;4(3):691–705. doi: 10.1158/2767-9764.CRC-23-0396 (PMC10919208; doi:10.1158/2767-9764.CRC-23-0396)

**Figure S2.** Brightfield images of cell lines after treatment with LD_50_ cisplatin. Cells were plated into 6 well dishes and exposed to cisplatin for 72 h with a minimum of three technical replicates per concentration with 3 biological replicates performed. Cells were washed with PBS prior to images taken in brightfield with Olympus microscope at 10x magnification.


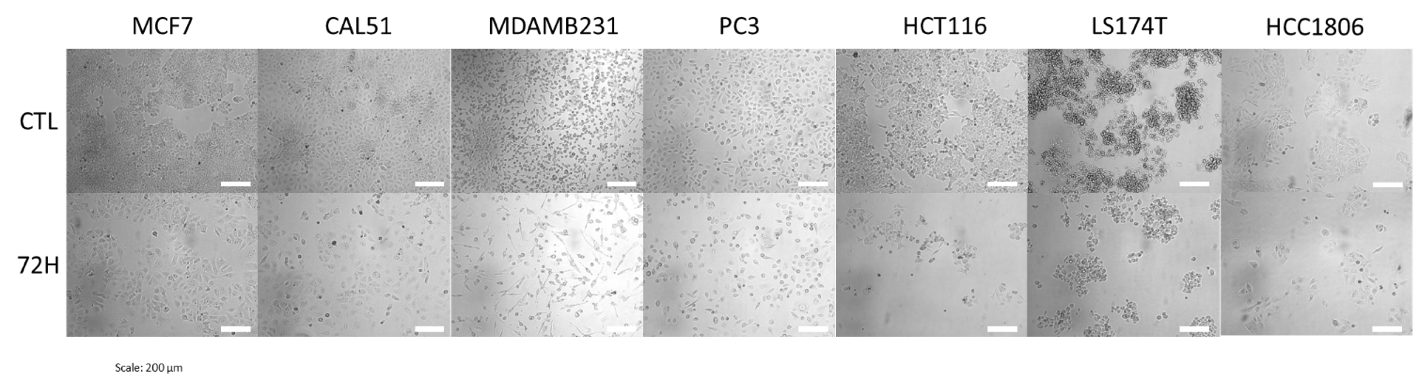

Supplement: Figure S2 — Brightfield images of cell lines after treatment with LD50 cisplatin. [file crc-23-0396-s10.docx]
