# Supplementary material for: Drug-resilient Cancer Cell Phenotype Is Acquired via Polyploidization Associated with Early Stress Response Coupled to HIF2α Transcriptional Regulation
Source: Cancer Res Commun. 2024 Mar 7;4(3):691–705. doi: 10.1158/2767-9764.CRC-23-0396 (PMC10919208; doi:10.1158/2767-9764.CRC-23-0396)
Supplement: Figure S7 — Drug-resilient 786-0 cells exhibit 1-2 whole genome duplications with high fidelity [file crc-23-0396-s15.docx]

**Figure S7**. Drug-resilient 786-0 cells exhibit 1-2 whole genome duplications with high fidelity


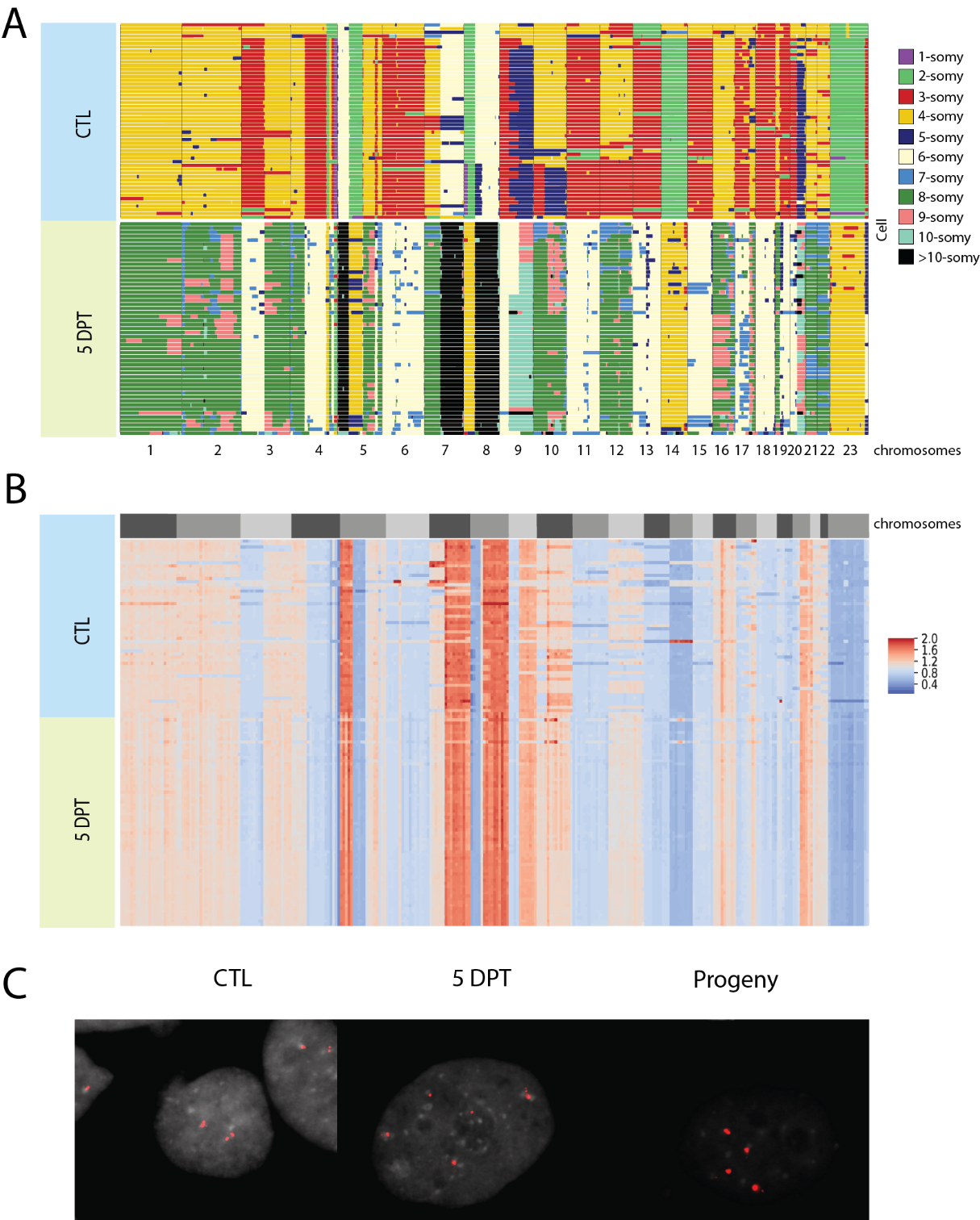


Aneufinder analysis was performed on 786-0 cells for CTL and 5 DPT cells. Cells at 5 DPT had undergone multiple whole genome duplications (A). Normalised read counts of 786-0 CTL and 5 DPT scWGS (B). Copy number of chromosome X in untreated (CTL), surviving cells at 5 DPT and their progeny, as visualized with FISH of cells in interphase (C).
