# Supplementary material for: Drug-resilient Cancer Cell Phenotype Is Acquired via Polyploidization Associated with Early Stress Response Coupled to HIF2α Transcriptional Regulation
Source: Cancer Res Commun. 2024 Mar 7;4(3):691–705. doi: 10.1158/2767-9764.CRC-23-0396 (PMC10919208; doi:10.1158/2767-9764.CRC-23-0396)
Supplement: Figure S8 — Pathways downregulated in HCC1806 cells surviving 10 DPT, as quantified with RNAseq and Reactome analysis. [file crc-23-0396-s16.docx]

**Figure S8.** Pathways downregulated in HCC1806 cells surviving 10 DPT, as quantified with RNAseq and Reactome analysis.


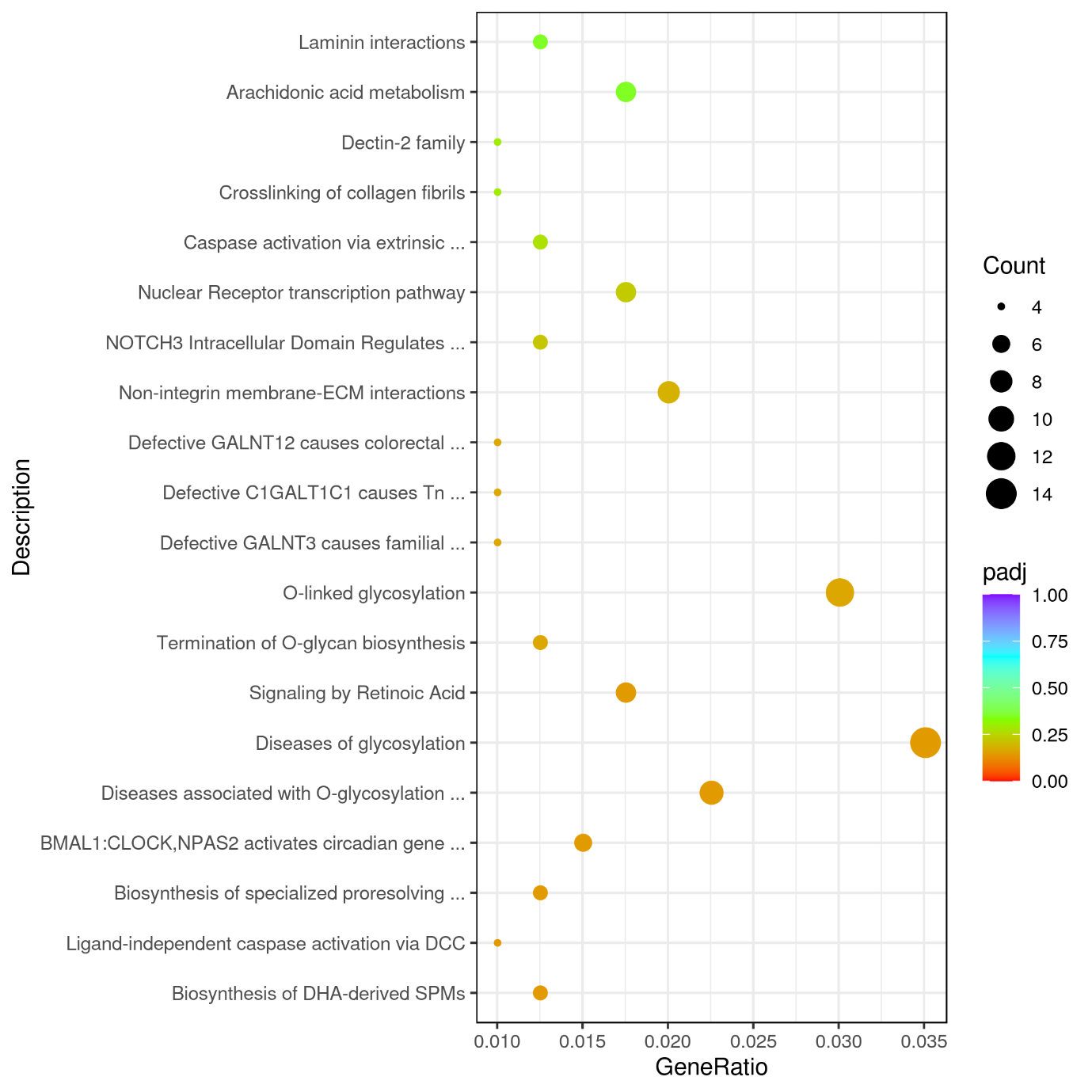


Reactome analysis was performed on HCC1806 cells surviving at 10 DPT using the downregulated differentially expressed gene list. Mechanistic pathways which were frequently downregulated included: Glycosylation, metabolic pathways, ECM interactions.
