# Supplementary material for: Drug-resilient Cancer Cell Phenotype Is Acquired via Polyploidization Associated with Early Stress Response Coupled to HIF2α Transcriptional Regulation
Source: Cancer Res Commun. 2024 Mar 7;4(3):691–705. doi: 10.1158/2767-9764.CRC-23-0396 (PMC10919208; doi:10.1158/2767-9764.CRC-23-0396)
Supplement: Figure S9 — Transcription factors regulating downregulated genes in HCC1806 cells surviving 10 DPT as quantified using RNAseq and CHEA3 analysis. [file crc-23-0396-s17.docx]

**Figure S9**. Transcription factors regulating downregulated genes in HCC1806 cells surviving 10 DPT as quantified using RNAseq and CHEA3 analysis.

Transcription factor analysis was undertaken on downregulated HCC1806 at cells 10 DPT. For ChEA3 the following TF-target gene set libraries were assembled: putative targets as determined by ENCODE, ReMap; co-expression of TFs with other genes based on processed RNAseq from GTEx and ARCHS4; co-occurrence of TFs with other genes by examining gene lists submitted to the tool Enrichr; and gene signatures resulting from single TF perturbations followed by genome-wide gene expression experiments.
