# Supplementary material for: Drug-resilient Cancer Cell Phenotype Is Acquired via Polyploidization Associated with Early Stress Response Coupled to HIF2α Transcriptional Regulation
Source: Cancer Res Commun. 2024 Mar 7;4(3):691–705. doi: 10.1158/2767-9764.CRC-23-0396 (PMC10919208; doi:10.1158/2767-9764.CRC-23-0396)

**Figure S10.** EPAS1 FPKM reads at different timepoints post treatment in HCC1806. EPAS1 FPKM reads after different treatment modalities at 72h post treatment in HCC1806. EPAS1 FPKM reads in different cell lines at 72h post cisplatin treatment. Visualization of *EPAS1* gene expression of HCC1806 cells when untreated, surviving treatment at 0 DPT, 5 DPT, 10 DPT, and as progeny. Visualization of *EPAS1* gene expression of HCC1806 cells when untreated, treated with LD50 Doxorubicin or Vincristine for 72h, or 72h after ionizing Radiation treatment. Visualization of *EPAS1* gene expression of 786-0, U1690, and HCT116 cell after 72h cisplatin treatment. Expression taken from FKPM reads of RNAseq analysis.


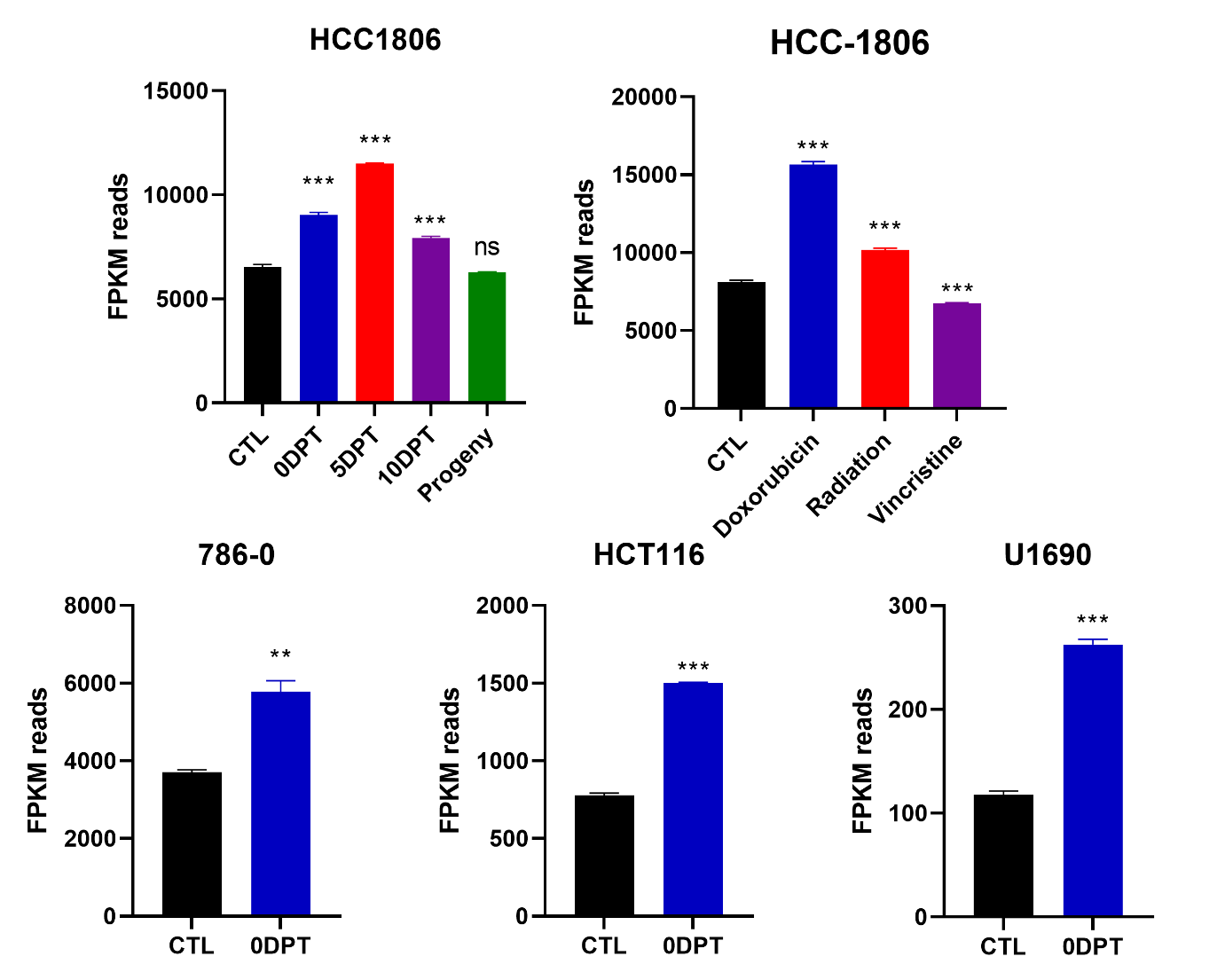

Supplement: Figure S10 — EPAS1 FPKM reads at different timepoints post treatment in HCC1806. [file crc-23-0396-s18.docx]
