# Supplementary material for: Drug-resilient Cancer Cell Phenotype Is Acquired via Polyploidization Associated with Early Stress Response Coupled to HIF2α Transcriptional Regulation
Source: Cancer Res Commun. 2024 Mar 7;4(3):691–705. doi: 10.1158/2767-9764.CRC-23-0396 (PMC10919208; doi:10.1158/2767-9764.CRC-23-0396)

**Figure S12.** Survival of polyploid cells is reduced by the addition of NOTCH inhibitor during cisplatin treatment. Number of HCC1806, HCT116, and 786-0 cells surviving at 0 DPT and 10 DPT when treated with cisplatin only and cisplatin together with PF-03084014 (inhibiting NOTCH signaling). Three biological replicates, ∗∗∗p < 0.001, ∗∗p < 0.01, ∗p < 0.05.


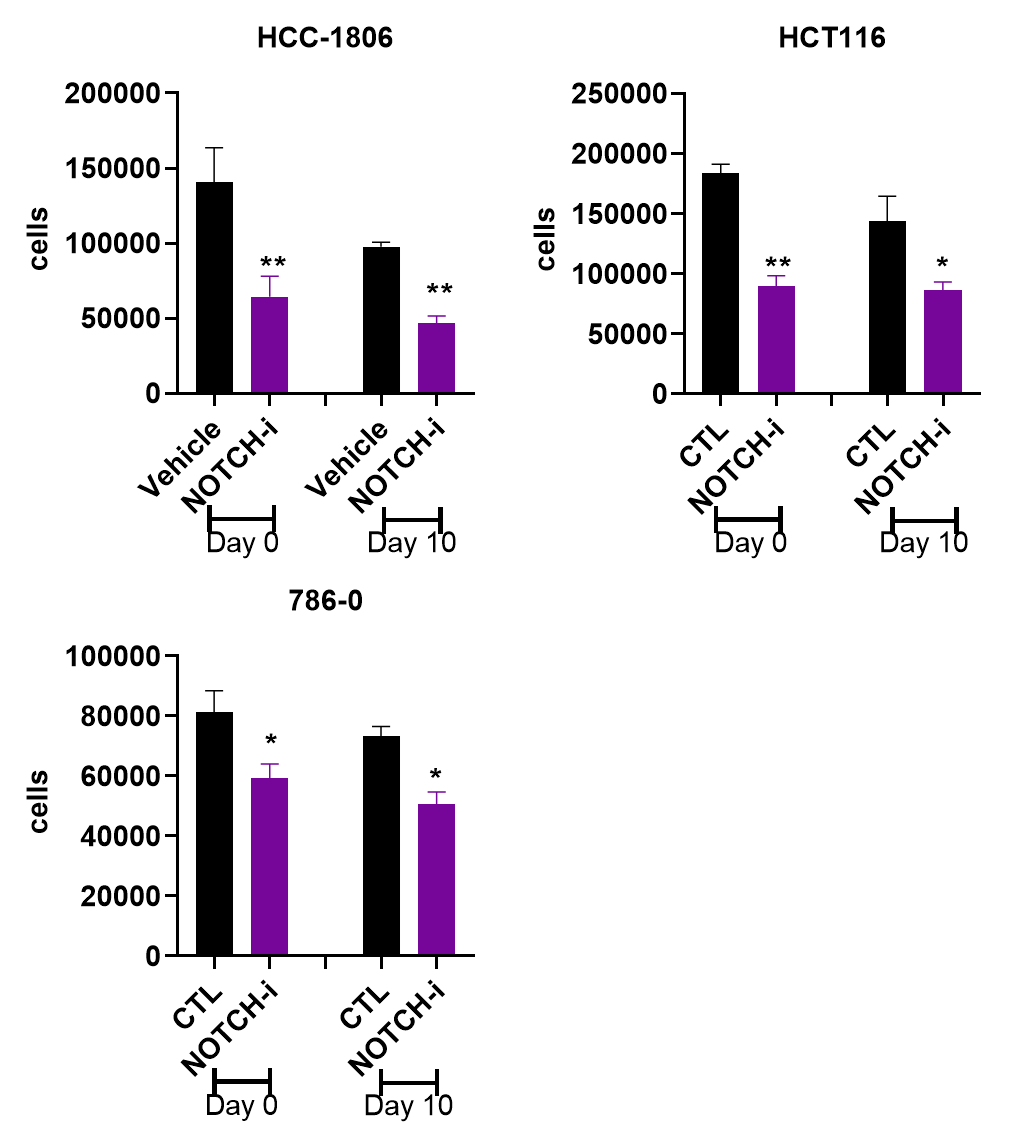

Supplement: Figure S12 — Survival of polyploid cells is reduced by the addition of NOTCH inhibitor during cisplatin treatment. [file crc-23-0396-s20.docx]
