# Supplementary material for: Drug-resilient Cancer Cell Phenotype Is Acquired via Polyploidization Associated with Early Stress Response Coupled to HIF2α Transcriptional Regulation
Source: Cancer Res Commun. 2024 Mar 7;4(3):691–705. doi: 10.1158/2767-9764.CRC-23-0396 (PMC10919208; doi:10.1158/2767-9764.CRC-23-0396)
Supplement: Figure S13 — Effects of T2445 treatment on cellular proliferation at 10nM. [file crc-23-0396-s21.docx]

**Figure S13.** Effects of T2445 treatment on cellular proliferation at 10nM. Treatment with T2445 did not inhibit proliferation in HCC1806 nor HCT116. Three biological replicates were tested, ∗∗∗p < 0.001, ∗∗p < 0.01, ∗p < 0.05.

**
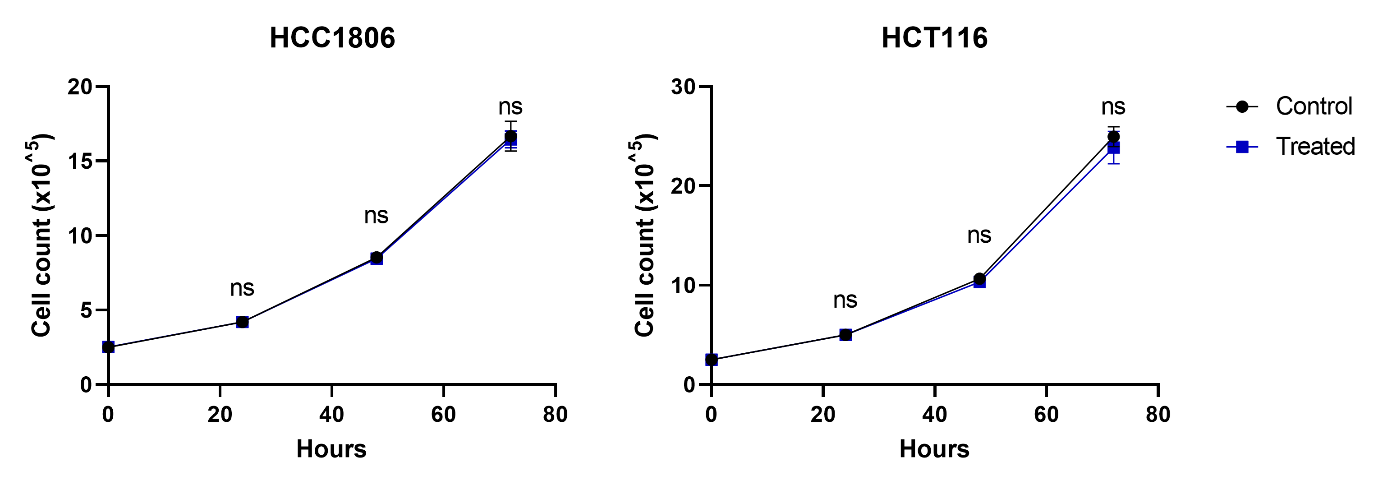
**
